# Supplementary material for: Turning challenges into opportunities: Lessons from Ethiopia’s COVID-19 response for strengthening health systems and health security
Source: PLOS Glob Public Health. 2025 Aug 20;5(8):e0005052. doi: 10.1371/journal.pgph.0005052 (PMC12367127; doi:10.1371/journal.pgph.0005052)
Supplement: S1 File — (DOCX) [file pgph.0005052.s001.docx]

**Reported Barriers to Ethiopia’s COVID-19 Response**

| **Barrier** | **Number** | **%** | **CFIR domain** |
| --- | --- | --- | --- |
| **Major surveillance related barriers at the early phase of the pandemic as reported by the quantitative survey respondents (n = 396)** | | |  |
| Inadequate capability (Knowledge, attitudes, skills, and self-efficacy)  of surveillance staff | 52 | 13.1 | Individual characteristics |
| Inadequate surveillance related internal policies/norms | 33 | 8.3 | Inner setting |
| Inadequate teamwork/coordination among surveillance staff across  the country | 27 | 6.8 | Inner setting |
| Lack of strong disease surveillance culture | 41 | 10.4 | Inner setting |
| Inadequate rewards and motivation of surveillance staff | 63 | 15.9 | Inner setting |
| Inadequate existing organizational commitment for surveillance | 46 | 11.6 | Inner setting |
| Surveillance approach was not well adapted to various contexts of the  country | 62 | 15.7 | Program characteristics |
| Complexity of surveillance procedures and tools | 85 | 21.5 | Program characteristics |
| Not adequately supported by finance and other logistics | 76 | 19.2 | Outer Setting |
| Social barriers (resistance to report suspected cases, denial, stigma) | 60 | 15.2 | Outer Setting |
| Lack of intermate and phone line connectivity | 36 | 9.1 | Outer Setting |
| **Major lab testing related barriers at the early phase of the pandemic as reported by the quantitative survey respondents (n= 208 )** | | |  |
| Inadequate capability (Knowledge, attitudes, skills, and self-efficacy)  of laboratory staff | 52 | 25.0 | Individual characteristics |
| Inadequate physical structure or space for laboratory testing | 65 | 31.3 | Inner Setting |
| Inadequate referral system for testing | 53 | 25.5 | Inner Setting |
| Lack of standards/norms for sample processing and reporting | 33 | 15.9 | Implementation process |
| Inadequate teamwork/coordination among surveillance, lab testing and  case management staff | 36 | 17.3 | Inner Setting |
| Lack of rewards and motivation of lab staff | 63 | 30.3 | Inner Setting |
| Lack of adequate resources (manpower, safety gears, finance, reagents,  equipment) | 46 | 22.1 | Inner Setting |
| Complexity of lab procedures including sample collection, transport,  processing, and reporting | 85 | 40.9 | Program characteristics |
| **Major case management related barriers at the early phase of the pandemic as reported by the quantitative survey respondents (n= 210 )** | | |  |
| Inadequate capability (Knowledge, attitudes, skills, and self-efficacy) of  case management staff | 86 | 41.0 | Individual characteristics |
| Inadequate infrastructure, including convenient and well equipped  space for critical care management | 85 | 40.5 | Inner setting |
| Weak patient referral system | 57 | 27.1 | Inner setting |
| Lack of rewards and motivation to case management team | 63 | 30.0 | Inner setting |
| Lack of life saving equipment and devices | 44 | 21.0 | Inner setting |
| Complexity in patient admission, management, and discharge  procedures | 96 | 45.7 | Program characteristics |
| Lack of proper planning, supervision and feedback | 62 | 29.5 | Implementation process |
| Social cultural barriers (case management centers were  stigmatized) | 70 | 33.3 | Outer setting |
| **Major IPC related barriers at the early phase of the pandemic as reported by the quantitative survey respondents (n = 471)** | | |  |
| Inadequate capability (Knowledge, attitudes, skills, and self-efficacy)  of IPC staff and the general public | 138 | 29.3 | Individual characteristics |
| Inconvenient facility structures/designs for IPC within the health care  setting | 131 | 27.8 | Inner setting |
| Inadequate IPC guides, norms, and standards at different levels | 56 | 11.9 | Inner setting |
| Fragmented coordination and public panic (fear and anxiety) | 44 | 9.3 | Outer setting |
| Inadequate IPC gears | 69 | 14.6 | Inner setting |
| Economic related (the public did not comply with quarantine measures  due to economic hardship) | 183 | 38.9 | Outer setting |
| Socio-cultural barriers | 213 | 45.2 | Outer setting |
| **Major vaccination related barriers at the early phase of the pandemic as reported by the quantitative survey respondents (n = 533)** | | |  |
| Inadequate capability (Knowledge, attitudes, skills, and self-efficacy) of  vaccination staff and the general public | 185 | 34.7 | Individual characteristics |
| Inadequate vaccine facilities (cold chain and other items) | 74 | 13.9 | Inner setting |
| Lack of coordination between the vaccination team (EPI team) and  other COVID-19 response team | 28 | 5.3 | Inner setting |
| Inadequate rewards and motivation to the vaccine team | 64 | 12.0 | Inner setting |
| Lack of confidence on the vaccine | 99 | 18.6 | Outer setting |
| Strategy complexity (keeping social distancing vs administrating the  vaccine in large meetings) | 156 | 29.3 | Program characteristics |
| Communication not tailored to various contexts | 164 | 30.8 | Inner setting |
| Political barriers/vaccine nationalism | 51 | 9.6 | Outer setting |
| Socio-cultural barriers (hesitancy as a result of cultural or  religious beliefs) | 272 | 51.0 | Outer setting |
