# Supplementary material for: Turning challenges into opportunities: Lessons from Ethiopia’s COVID-19 response for strengthening health systems and health security
Source: PLOS Glob Public Health. 2025 Aug 20;5(8):e0005052. doi: 10.1371/journal.pgph.0005052 (PMC12367127; doi:10.1371/journal.pgph.0005052)
Supplement: S4 File — (DOCX) [file pgph.0005052.s004.docx]

**National Assessment of COVID-19 Response in Ethiopia: experiences, Lessons learned and future directions.**

**Structured questionnaire – individuals survey**

Thank you for your participation in this study. You have been identified as a member of the COVID-19 response team and we are interested in hearing about your experiences working to contain the pandemic. Your insights will be used to inform an understanding of the experiences and lessons learned from the COVID-19 response that could be applied to future pandemic preparedness and response. This survey will take up to 40 minutes to complete depending on numbers of COVID-19 response pillars you were involved with. Your personal information and responses will remain confidential and will not be shared beyond the research team without your consent.

| **No.** | **Question** | **Response** | **Skip to** | |
| --- | --- | --- | --- | --- |
| **Section 1 - Identifiers** | | |  | |
| 100 | Individual Code |  |  | |
| 101 | Organization code  *(For health facilities, organization code is the same as facility code)* |  |  | |
| 102 | Region | 1 Amhara  2 Oromia  3 Somali  4 Gambella  5 Addis Ababa  6 Central |  | |
| 103 | Zone (lists to be programed in ODK) | _________________ |  | |
| 104 | Woreda (lists to be programed in ODK) | _________________ |  | |
| 105 | Organization name | _________________ |  | |
| 106 | Type of organization | 1 Ministry of health  2 Agency of MOH  3 Professional association  4 Implementing partner  5 Regional health bureau  6 Regional public health institute  7 Regional EPSS hub  8 Regional implementing partner  9 Woreda health office  10 Hospital  11 Health center  12 health post  13 Other (specify-----) |  | |
| 107 | Geographic coordinate (organization location) | North -----------------  East ------------------- |  | |
| **Section 2 - Respondent Characteristics** | | | | |
| 201 | Age  *(Age should be ≥ 18 or ≤ 80)* |  |  | |
| 202 | Sex of respondent | 1 Male  2 Female |  | |
| 203 | Educational status of respondent | 1 Certificate  2 first Degree  3 Medical degree (including specialty certificate)  4 Second Degree  5 Third Degree |  | |
| 204 | Which pillars of the COVID-19 pandemic response have you ever been involved in?  *(Please circle all that apply)* | 1 Surveillance and outbreak investigation  2 Laboratory testing and diagnostics  3 Facility preparedness and case management  4 Infection prevention and control  5 Risk communication and community engagement  6 Logistics and supply chain  7 Vaccination  8 Points of entry, international travel and mass gatherings  9 Maintaining essential health services during the COVID-19 pandemic | |  |
| 205 | Which description best defines your role(s) in the implementation of the COVID-19 response activities?  *(Please circle all that apply)* | 1 Policy Maker  2 COVID-19 program lead/coordinator.  3 Technical Advisor  4 Researcher  5 Incident manager  6 EPI Manager/Coordinator  7 Supply chain manager/officer  8 Resource mobilization coordinator/Officer  9 Facility director/head/CEO  10 Facility COVID-19 surveillance focal person  11 Pharmacist  12 Facility EPI/Immunization focal person  13 Laboratory personnel  14 Health extension worker  15 Communication manager/officer  16 Other (Specify)----------- | | |
| 206 | Of the previously listed roles (Q205), which one did you hold for the longest period?  (Please select the best answer)  If more than one response is selected at Q205 | 1 Policy Maker  2 COVID-19 program lead/coordinator.  3 Technical Advisor  4 Researcher  5 Incident manager  6 EPI Manager/coordinator  7 Supply chain manager/officer  8 Resource Mobilization Officer  9 Facility director/head/CEO  10 Facility COVID-19 surveillance focal person  11 Pharmacist  12 Facility EPI/immunization focal person  13 Laboratory personnel  14 Health Extension worker  15 Communication manager/officer  16 Other (Specify)----------- |  | |
| 207 | During what years did you perform the role of (choice from Q205) | - Start- month and year ______________ - End - month and year _______________ |  | |
| 208 | At which phase of the pandemic you were engaged in the response?  Please check all that apply. | 1 Early Response (March to September 2020)  2 Response (October 2020 to December 2021)  3 Mid-Response (January 2022 to September 2022)  4 Late Response (October 2022 to Present) |  | |

| B. Surveillance, rapid response teams and case investigation (Check Q204 and s*kip this section if the respondent was not involved in this pillar*) | | | |
| --- | --- | --- | --- |
| ***To the respondent:*** For this next set of questions, we would like to ask you about implementation facilitators and challenges relevant to the pillar of Surveillance.  ***To the data collector:*** *Please make sure that the respondent understands each of the questions. If you need help to explain each question to the respondent, please click the buttons below each question in your device. A detailed explanation will pop up.* | | | |
|  | Question | Response | Detailed explanations of each of the responses |
| ***The next five questions ( B401 to B405) are about the facilitators or contributors of the COVID-19 surveillance activities in your organization.*** | | | |
| B401 | Which of the following were the internal contributors to your success in the COVID-19 surveillance, rapid response teams and case investigations pillar?  (Please check all that apply) | 1 Knowledge, skills, attitude, and self-efficacy of individuals within your organization involved in the COVID-19 surveillance and case investigation.  2 Conducive organizational setting    3 Simplicity, feasibility, acceptability, and applicability of the COVID-19 surveillance approaches  4 Well-planned and well-coordinated process of the COVID-19 response activities | The first option refers to the knowledge (familiarity with facts, truths and principles related to the COVID-19 related surveillance approaches including knowledge of case definitions, case investigation procedures, case isolation procedures, reporting procedures and quarantine procedures), self-efficacy (belief in one’s own abilities to execute required courses of action) of surveillance personnel, and skills of surveillance personnel (how likely or not the individual is to provide skilled, enthusiastic and sustained support of the COVID-19 surveillance activities throughout the different stages of the response), the attitude of surveillance personnel towards COVID-19 surveillance, motivation to conduct COVID-19 surveillance, etc).  The second option refers to factors related to your organization supporting the COVID-19 response. These include conducive atmosphere within the organization to conduct COVID-19 surveillance such as strong work ethic, strong teamwork, robust coordination within the organization, robust leadership within the organization to conduct surveillance, accountability mechanisms within the organization, the organization’s commitment to provide support to the surveillance program in terms of finance, supplies, tools, and equipment, etc. in a timely manner.  The third option refers to the COVID-19 surveillance program itself. This includes the simplicity and feasibility of the surveillance approach including technologies and tools used such as guidelines, manuals, and case investigation forms adapted by the organization and the simplicity, applicability, and feasibility of these tools. It also includes approaches and strategies used to conduct surveillance such as contact tracing, case investigation, isolation, quarantine, etc.  The fourth option refers to the process of how surveillance activity was implemented, including the planning (implementation schemes/methods in advance), execution strategies (carrying out activities according to plan), engaging (attracting and involving appropriate stakeholders in implementation) reflection and evaluation of activities (monitoring program progress and quality, including regular debriefing about progress and experience) , or adjustments made to the plan. |
| B402 | Among the internal contributors selected at B401, if you rank based on the magnitude of contribution, which one is the most significant contributor?  (Skip this question if only one choice is checked at QB401) | 1 Knowledge, skills, attitude, and self-efficacy of individuals within your organization involved in the COVID-19 surveillance.  2 Conducive organizational setting    3 Simplicity, feasibility, acceptability, and applicability of the COVID-19 surveillance approaches  4 Well planned and well-coordinated process of the COVID-19 response activities | Please see the explanations above. |
| B403 | Which of the following were the external contributors to your COVID-19 surveillance success?  (Please check all that apply) | 1 Conducive political environment    2 Supportive economic environment  3 Conducive social environment    4 Supportive technological environments  5 Other environment (Please describe). | The first option refers to parliament/regional council support, political climate acceptance of the COVID-19 surveillance activities (high political will), policies and legal provisions are adequate to conduct surveillance, and political structure including peace and security are conducive to conducting surveillance.  The second option refers to the availability of sufficient revenue sources and budget to fund surveillance activities and/or maintain surveillance system developments.  The third option refers to changing social norms and social support around COVID-19 prevention and surveillance, and conducive and accepting communities for COVID-19 surveillance, and case investigation.  The fourth option refers to technological advances outside of the organization during the time of the pandemic that could be harnessed for COVID-19 surveillance. For example, telecommunication networks are conducive to reaching populations, electronic platforms are available to record and report real-time data, technologies are available to support case investigation, etc. |
| B404 | Among the external contributors selected at B403, if you are asked to rank based on the magnitude of contribution, which one is the most significant contributor?  (Skip this question if only one choice is checked at QB403) | 1 Conducive political environment    2 Supportive economic environment  3 Conducive social environment  4 Supportive technological environments  5 Other environment (Please describe). | Please see the explanations at question # B403 |
| B405 | Please briefly describe the most influential internal, external or combination of contributors to your program's success in completing the surveillance, rapid response teams, and case investigation pillar | -------------------------------------------------------------------------------------------------------------------------------------------------------------------------------------------------------------------------- | Ask the respondent to describe in her/his own words the most significant contributors of the surveillance program. Please write the responses verbatim. |
| ***The following questions are about the barriers to COVID-19 surveillance. First, I will ask you (Question # B406) about the overall challenges related to the surveillance program. Next (Questions from B407 to B410), I will ask about the barriers related to the individuals who implemented surveillance, challenges related to your organization, challenges related to the surveillance approach itself, the processes of implementation, and the barriers external to your organization. I will ask you all these questions one by one.*** | | | |
| B406 | Which of the following best describes the key challenges when carrying out activities associated with COVID-19 surveillance, rapid response teams and case investigation?  (Please check all that apply) | 1 Challenges related to the knowledge, skills, attitude, and self-efficacy of individuals within your organization involved in COVID-19 surveillance.    2 Challenges related to your organization settings.  3 Challenges related to the complexity, applicability, and acceptability of the COVID-19 surveillance approaches and activities.  4 Challenges related to the planning, supervision, engagement, and monitoring of the surveillance activities. | The first response refers to challenges related to the lack of knowledge of surveillance personnel on COVID-19 surveillance basics and facts. These include knowledge of case definitions and case investigation procedures, and how cases are identified, isolated, reported, and quarantined. It also includes challenges related to the lack of self-efficacy of surveillance personnel, attitude of surveillance personnel towards COVID-19 surveillance, lack of motivation to conduct COVID-19 surveillance, resistance to change among surveillance personnel, etc.  The second option refers to challenges related to factors in the organization supporting the COVID-19 response. These include a challenging atmosphere within the organization to conduct surveillance such as lack of structures and space, lack of internal policies, lack of work ethic in the organization, lack of teamwork, lack of robust disease surveillance culture in the organization, lack of use of innovation in the organization, lack of rewards and motivation policies including staff development, lack of robust coordination and leadership within the organization, lack of organization commitment to providing support to the surveillance program in terms of finance, supplies, tools, and equipment, etc.  The third option refers to challenges related to the surveillance program design. This includes the complexity and lack of feasibility of the surveillance program, including technologies and tools used such as guidelines, manuals, and case investigation forms adapted by the organization. Surveillance tools lack applicability and feasibility. It also includes complex and non-simplistic approaches and strategies used to conduct surveillance such as contact tracing, case investigation, isolation, quarantine, etc.  The fourth option refers to challenges related to how the activity was implemented, including lack of planning, lack of execution strategies as planned, lack of reflection and evaluation of activities, or lack of adjustment of the plan in the changing context.  The fifth option refers to low political will, insecurity and conflict, insufficient funding or limited resources, community resistance to surveillance activities, lack of conducive technology or technology not friendly, etc. |
| B407 | What characteristic(s) best describes implementation challenges you encountered while conducting COVID-19 surveillance related to an individual’s characteristics?  (Please check all that apply)  Skip this if it is not selected in question # B406 | 1 Surveillance personnel’s lack of knowledge about the activity  2 Surveillance personnel negative attitudes and beliefs about the COVID-19 surveillance activity  3 Surveillance personnel lack the skills to conduct the activity.  4 Surveillance personnel lack self-efficacy to conduct the activity  5. Health worker burnout  6 Other personal attributes (please describe) | The first option refers to surveillance personnel who were unfamiliar with facts, truths and principles related to COVID-19 surveillance including case definitions, case investigation procedures, reporting procedures, etc.  The second option refers to surveillance personnel who did not have positive attitude toward the COVID-19 surveillance program, including the feeling that COVID-19 surveillance is not a priority.  The third option refers to how likely (or not) the surveillance personnel are to provide skilled, enthusiastic, and sustained support of the COVID-19 surveillance throughout the different phases of pandemic.  The fourth option refers to surveillance personnel’s lack of belief in her/his own abilities to execute required courses of action related to COVID-19 surveillance.  The fifth option refers to long-term stress reactions of surveillance personnel as a result of overload, long time work, etc, and the influence of burnout on the COVID-19 surveillance program. |
| B408 | What characteristic(s) best describes implementation challenges to COVID-19 surveillance you encountered related to your organizational setting?  (*Please check all that apply)*  Skip this if it is not selected in question # B406 | 1 Lack of physical structure and space to conduct surveillance within the organization.  2 Lack of strong internal and external referral systems  3 Lack of internal policies, norms, and values within the organization  4 Lack of work ethic in the organization  5 Lack of teamwork  6 Lack of strong disease surveillance culture in the organization    7 Lack of rewards and motivation in the organization  8 Lack of robust coordination and leadership  9 Organization lacks the commitment to provide resource for COVID-19 surveillance, rapid response teams and case investigation.  10 Other challenges attributed to the organization (please describe) | The first option refers to challenges related to physical infrastructure within the organization, information technology infrastructure (communications, electronic recording, data storage, and transfer, interoperability of electronic platforms), work infrastructure (staffing level, support staff, staff turnover), how the surveillance team is structured or organized, etc.  The second option refers to challenges related to internal referral systems, external referral systems, and communication between and among professionals.  The third option refers to challenges related to organizational norms and values. It includes institutional culture related to the use of evidence-based practice, shared mission and vision, lack of long-term experiences accumulated and recognized by the community it serves, lack of organizational readiness to adapt, relation with the community it serves, lack of rewarding policies and procedures, lack of accountability mechanisms, etc.  The eighth and ninth options refer to challenges related to a lack of leadership engagement, limited available resources, and poor access to knowledge and information for the surveillance staff. |
| B409 | What characteristic(s) best capture implementation challenges to COVID-19 surveillance you encountered related to COVID- 19 surveillance characteristics or the surveillance program itself?  (Please check all that apply)  Skip this if it is not selected in question # B406 | 1 Lack of acceptance of the surveillance design by the people who conduct surveillance.  2 The perception from the surveillance staff that the effectiveness of the surveillance approach or design is not tested systematically and lack evidence to implement it.  3 Complexity of surveillance tools and approaches  4 Not well adapted to local contexts.    5 Cost of the surveillance approach is expensive  6 Other (please describe) | The first option refers to the perception that the COVID-19 surveillance design is made by an external body (imposed from external bodies) and the perception that it is not appropriate for them.  The second option refers to the perceptions from the surveillance staff that the quality and validity of the evidence did not support that the surveillance design would have the desired outcomes.  The third option refers to the perceived difficulty of the COVID-19 surveillance approach. This may include: case definitions that are too complex to understand, complex case investigation processes, time taking recording and paperwork, etc.  The fourth option refers to COVID-19 surveillance was not adapted, tailored, or refined to meet local needs. Eg, community surveillance was not adapted to use informal structures within the community.  The fifth option refers to the cost of surveillance-related activities including intervention, supply, and opportunity costs. The cost-effectiveness and the cost-benefits of the surveillance approach seem inappropriate. |
| B410 | What characteristic(s) capture implementation challenges of COVID-19 surveillance you encountered related to external settings?  Please check all that apply  Skip this if it is not selected in question # B406 | 1 Political environment  2 Economic environment    3 Social environment  4 Technological environments.  5 Other environment | The first option refers to policymaker disinterest or resistance to surveillance activities, limited windows of opportunity within the political climate, and political structure non-conducive to coordinated action, conflict, and unrest.  The second option refers to insufficient revenue sources to fund surveillance activities and/or maintain surveillance system developments.  The third option refers to communities in which COVID-19 response activities were implemented that are non-accepting and/or resistant to COVID-19-related surveillance, including case identification, isolation, case investigation, etc.    The fourth option refers to slow or limited advances of technologies used in implementing COVID-19 response activities, including communication technology and coverage of mobile and internet services, electronic recording and transfer of data, unavailability of case investigation technologies, etc.  The fifth option refers to the environment where the activity was implemented that was prohibitive and did not contribute to the success of the COVID-19 pandemic response, including ineffective cross-organizational collaboration. |
| B411 | Please describe the most influential internal, external or combination of challenges you experienced when carrying out activities associated with COVID-19 surveillance, rapid response teams and case investigation pillar. | ---------------------------------------------------------------------------------------------------------------------------------------------------------------------------------------------------------------------------------------------------------------------------------------------------------------------------------------------------------------------------------- | Ask the respondent to describe in her/his own words the most significant challenges to the surveillance program. Please write the responses verbatim. |
| B412 | At what stage(s) of the process of COVID-19 response implementation did you experience the most implementation challenges related to surveillance?  (Please check all that apply) | 1 Early Response (March to September 2020)  2 Response (October 2020 to December 2021)  3 Mid-Response (January 2022 to September 2022)  4 Late Response (October 2022 to Present) |  |
| B413 | What were the activities you conducted to overcome these challenges? | 1)____________  2)____________  3)____________  4)____________ |  |

| C. Laboratory testing and diagnostics (*Check Q204 and skip this section if the respondent was not involved in this pillar*) |
| --- |

| ***To the respondent:*** For this next set of questions, we would like to ask you about implementation facilitators and challenges relevant to the pillar of laboratory testing and diagnostics.  ***To the data collector:*** Please note any contradictions in the responses (for example between C401 (facilitator or success) and C406 (challenge)) and ask explanation from the respondent before leaving the area where the data collection is undertaken. | | | |
| --- | --- | --- | --- |
|  | Question | Response | Detailed explanation of each of the responses |
| ***The next five questions ( C401 to C405) are about the facilitators or contributors of COVID-19 laboratory testing and diagnostics.*** | | | |
| C401 | Which of the following were the internal contributors to your success in the COVID-19 laboratory testing and diagnostics pillar?  *Please check all that apply.* | 1 Knowledge, skills, attitude, and self-efficacy of individuals within your organization involved in the COVID-19 testing.  2 Conducive organizational setting    3 Simplicity of the COVID-19 testing procedures  4 Well-planned and well-coordinated process of the COVID-19 testing activities | The first option refers to the knowledge (familiarity with facts, truths, and principles related to the COVID-19 lab testing approaches including knowledge on how and when to collect samples, testing procedures, transporting of lab samples, and reporting of test results), self-efficacy (belief in one’s own abilities to execute required courses of action) of lab personnel, and skills of lab personnel (how likely or not the individual is to provide skilled, enthusiastic and sustained support of the COVID-19 lab testing activities throughout the different stages of the response), the attitude of lab personnel towards COVID-19 testing, motivation to conduct COVID-19 testing, etc).  The second option refers to factors related to your organization supporting the COVID-19 response. These include a conducive atmosphere within the organization to conduct COVID-19 testing such as a strong work ethic, strong teamwork, robust coordination within the organization, robust leadership within the organization to conduct testing, accountability mechanisms within the organization, the organization’s commitment to providing support to the lab testing program in terms of finance, supplies, tools, and equipment, etc. in a timely manner.  The third option refers to the COVID-19 lab testing program itself. This includes the simplicity of the case management approach including technologies and tools used such as guidelines, and manuals adapted by the organization, and the simplicity, applicability, and feasibility of these tools. It also includes approaches and strategies used to conduct lab testing.  The fourth option refers to the process of how testing activity was implemented, including the planning (implementation schemes/methods in advance), execution strategies (carrying out activities according to plan), engaging (attracting and involving appropriate stakeholders in implementation) reflection, and evaluation of activities (monitoring program progress and quality, including regular debriefing about progress and experience) , or adjustments made to the plan. |
| C402 | Among the internal contributors selected at QC401, if you are asked to rank based on the magnitude of contribution, which one is the most significant contributor?  Skip this if only one contributor is selected at QC401 | 1 Knowledge, skills, attitude, and self-efficacy of individuals within your organization involved in the COVID-19 lab testing.  2 Conducive organizational setting    3 Simplicity, feasibility, acceptability, and applicability of the COVID-19 lab testing approaches  4 Well planned and well-coordinated process of the COVID-19 lab testing activities | Please see the explanations above. |
| C403 | Which of the following were the external contributors to your COVID-19 lab testing success?  Please check all that apply | 1 Conducive political environment    2 Supportive economic environment  3 Conducive social environment    4 Supportive technological environments  5 Other environment (Please describe). | The first option refers to the political climate acceptance of the COVID-19 testing activities (high political will), policies and legal provisions are adequate to conduct lab testing, and political structure including peace and security are conducive to conduct l.  Ab testing in the area  The second option refers to the availability of sufficient revenue sources and budget to fund lab testing activities and/or maintain laboratory system developments.  The third option refers to changing social norms and social support around COVID-19 lab testing, and conducive and accepting communities for COVID-19 lab testing.  The fourth option refers to technological advances outside of the organization during the time of the pandemic that could be harnessed for COVID-19 testing. For example, telecommunication networks are conducive to reaching populations and informing test results, electronic platforms are available to record and report lab results, technologies are available to support seamless testing, etc. |
| C404 | Among the external contributors selected at QC403, if you are asked to rank based on the magnitude of contribution, which one is the most significant contributor?  Skip this if only one contributor is selected at QC403 | 1 Conducive political environment    2 Supportive economic environment  3 Conducive social environment  4 Supportive technological environments  5 Other environment (Please describe). | Please see the explanations in question # C403 |
| C405 | Please briefly describe the most influential internal, external or combination of contributors to your program's success in completing the lab testing and diagnostics pillar | -------------------------------------------------------------------------------------------------------------------------------------------------------------------------------------------------------------------------- | Ask the respondent to describe in her/his own words the most significant contributors of the lab testing and diagnostics pillar. Please write the responses verbatim. |
| ***The following questions are about the barriers to COVID-19 lab testing. First, I will ask you (Question # C406) about the overall challenges related to lab testing. Next (Questions from C407 to C410), I will ask about the barriers related to the individuals who implemented lab testing, challenges related to your organization, challenges related to the lab testing approach itself, the processes of implementation, and the barriers external to your organization. I will ask you all these one by one.*** | | | |
| C406 | Which of the following best describes the key challenges when carrying out activities associated with COVID-19 lab testing?  (Please check all that apply) | 1 Challenges related to the knowledge, skills, attitude, and self-efficacy of individuals within your organization involved in the COVID-19 lab testing.    2 Challenges related to your organization settings.  3 Challenges related to the complexity of the COVID-19 testing approaches and processes.  4 Challenges related to the planning, supervision, engagement, and monitoring of the testing process and activities.  5 Challenges related to the external settings, such as the political, technological, social and economic environment. | The first response refers to challenges related to the lack of knowledge of lab personnel on COVID-19 testing basics and facts. These include knowledge of how and what to test. It also includes challenges related to the lack of self-efficacy of lab personnel, attitude of lab personnel towards COVID-19 testing, lack of motivation to conduct COVID-19 testing, resistance to change among lab personnel, etc.  The second option refers to challenges related to factors in the organization supporting COVID-19 lab testing. These include a challenging atmosphere within the organization to conduct lab testing such as lack of structures and space, lack of internal policies, lack of work ethic in the organization, lack of teamwork , lack of quality control culture in the organization, lack of rewards and motivation policies including staff development, lack of robust coordination and leadership within the organization, lack of organization commitment to providing support to the lab testing process in terms of finance, supplies, tools, and equipment, etc.  The third option refers to challenges related to the lab testing approach. This includes the complexity and lack of feasibility of the testing process, including technologies and tools used such as guidelines and manuals adapted by the organization. Lab testing devices, supplies, and tools lack applicability and feasibility. It also includes complex and non-simplistic approaches in the testing process.  The fourth option refers to challenges related to how the activity was implemented, including lack of planning, lack of execution strategies as planned, lack of reflection and evaluation of lab activities, or lack of adjustment of the plan in the changing context.  The fifth option refers to low political will, insecurity, and conflict, insufficient funding or limited resources, community resistance to lab testing, lack of conducive technology or technology not friendly, etc. |
| C407 | What characteristic(s) best describes implementation challenges you encountered while conducting COVID-19 testing related to an individual’s characteristics?  (*Please check all that apply*)  Skip this if it is not selected in question # C406 | 1 Lab personnel lacks knowledge about the activity  2 Lab personnel attitudes and beliefs about the COVID-19 lab testing  3 Lab personnel lacks the skills to conduct lab testing (including collection, processing, and testing).  4 Lab personnel lacks self-efficacy  5. Lab personnel burnout  6 Other personal attributes (please describe) | The first option refers to lab personnel who were unfamiliar with facts, truths and principles related to COVID-19 lab testing including specimen collection procedure, processing and testing, etc.  The second option refers to lab personnel who did not have positive attitude toward the available COVID-19 lab testing, including the feeling that COVID-19 testing is not appropriate.  The third option refers to how likely (or not) the lab personnel are to provide skilled, enthusiastic, and sustained support of the COVID-19 testing throughout the different phases of pandemic.  The fourth option refers to lab personnel’s lack of belief in her/his own abilities to execute required courses of action related to COVID-19 lab testing.  The fifth option refers to long-term stress reactions of lab personnel as a result of overload, long time work, etc, and the influence of the burnout on the COVID-19 testing outcomes. |
| C408 | What characteristic(s) best describes implementation challenges to COVID-19 lab testing you encountered related to your organizational setting?  (*Please check all that apply)*  Skip this if it is not selected in question # C406 | 1 Lack of physical structure and space to conduct lab testing within the organization.  2 Lack of strong internal and external referral systems  3 Lack of internal policies, norms, and values within the organization  4 Lack of work ethic in the organization  5 Lack of teamwork  6 Lack of strong lab testing and quality control culture in the organization    7 Lack of rewards and motivation in the organization  8 Lack of robust coordination and leadership  9 Organization lacks the commitment to providing the resources for COVID-19 lab testing.  10 Other challenges attributed to the organization (please describe) | The first option refers to challenges related to physical infrastructure within the organization, information technology infrastructure (communications, electronic recording, data storage, and transfer, interoperability of electronic platforms), work infrastructure (staffing level, support staff, staff turnover), how the lab team is structured or organized, etc.  The second option refers to challenges related to internal referral systems, external referral systems, and communication between and among lab professionals and coordinators.  The third option refers to challenges related to organizational norms and values. It includes institutional culture related to the use of evidence-based practice, shared mission and vision, lack of long-term experiences accumulated and recognized by the community it serves, lack of organizational readiness to adapt, relation with the community it serves, rewarding policies and procedures, lack of accountability mechanisms, etc.  The eighth and ninth options refer to challenges related to lack of leadership engagement, limited available resources, and poor access to knowledge and information to the lab staff. |
| C409 | What characteristic(s) best capture implementation challenges to COVID-19 lab testing you encountered related to COVID- 19 lab testing characteristics or the lab testing approach itself?  (*Please check all that apply*)  Skip this if it is not selected in question # C406 | 1 Lack of acceptance of the lab testing approach by the people who conduct lab testing.  2 The perception from the lab staff that the effectiveness of the testing approach is not supported by evidence to implement it.  3 Complexity of testing procedures, tools and approaches  4 Not well adapted to local contexts.    5 Cost of the testing approach is expensive.  6 Other (please describe) | The first option refers to the perception that the COVID-19 testing approaches and procedure is made by an external body (imposed from external bodies) and the perception that it is not appropriate for them.  The second option refers to the perceptions from the lab staff that the quality and validity of the evidence did not support that the lab procedure would have the desired outcomes.  The third option refers to the perceived difficulty of the COVID-19 testing approach. This may include: procedures are too complex.  The fourth option refers to COVID-19 testing was not in line with local needs.  The fifth option refers to cost of lab testing including devices, supply, and opportunity costs. The cost effectiveness and the cost benefits of the COVID-19 testing seems inappropriate. |
| C410 | What characteristic(s) best capture implementation challenges of COVID-19 lab testing you encountered related to external settings?  *Please check all that apply*  Skip this if it is not selected in question # C406 | 1 Political environment  2 Economic environment    3 Social environment  4 Technological environment.  5 Other environment | The first option refers to policymaker disinterest or resistance to COVID-19 testing, limited windows of opportunity within the political climate, and a political structure non-conducive to coordinated action, conflict, and unrest.  The second option refers to insufficient revenue sources to fund COVID-19 testing and/or maintain lab system developments.  The third option refers to communities in which COVID-19 response activities were implemented that are non-accepting and/or resistant to COVID-19 testing.    The fourth option refers to slow or limited advances in technologies used in implementing COVID-19 testing.  The fifth option refers to the environment where the activity was implemented was prohibitive and did not contribute to the success of the COVID-19 pandemic response, including ineffective cross-organizational collaboration. |
| C411 | Please describe the most influential internal, external or combination of challenges you experienced when carrying out activities associated with the COVID-19 testing and diagnostics pillar. | ---------------------------------------------------------------------------------------------------------------------------------------------------------------------------------------------------------------------------------------------------------------------------------------------------------------------------------------------------------------------------------- | Ask the respondent to describe in her/his own words the most significant challenges to the COVID-19 lab testing pillar. Please write the responses verbatim. |
| C412 | At what stage(s) of the process of COVID-19 response implementation did you experience the most implementation challenges related to lab testing?  (Please check all that apply) | 1 Early Response (March to September 2020)  2 Response (October 2020 to December 2021)  3 Mid-Response (January 2022 to September 2022)  4 Late Response (October 2022 to Present) |  |
| C413 | What were the activities you conducted to overcome these challenges? | 1)____________  2)____________  3)____________  4)____________ |  |

| D. Facility preparedness and case management (*Check Q204 and skip this section if the respondent was not involved in this pillar*) |
| --- |

| ***To the respondent:*** For this next set of questions, we would like to ask you about implementation facilitators and challenges relevant to the pillar of facility preparedness and case management.  ***To the data collector:*** Please note any contradictions in the responses (for example between D401 (facilitator or success) and D406 (challenge)) and ask explanation from the respondent before leaving the area where the data collection is undertaken. | | | |
| --- | --- | --- | --- |
|  | Question | Response | Detailed explanation of each of the responses |
| ***The next five questions ( D401 to D405) are about the facilitators or contributors of COVID-19 case management.*** | | | |
| D401 | Which of the following were the internal contributors to your success in the COVID-19 case management pillar?  *Please check all that apply.* | 1 Knowledge, skills, attitude, and self-efficacy of individuals within your organization involved in the COVID-19 case management.  2 Conducive organizational setting    3 Simplicity of COVID-19 case management devices, guidelines, tools, protocols, etc  4 Well-planned and well-coordinated process of the COVID-19 case management activities | The first option refers to the knowledge of health providers (familiarity with facts, truths, and principles related to COVID-19 case management), self-efficacy of health providers (belief in one’s own abilities to execute required courses of action), and skills of health workers (how likely or not the individual is to provide skilled, enthusiastic and sustained support of the COVID-19 case management activities throughout the different stages of the response), attitude of health personnel towards COVID-19 case management, motivation to conduct COVID-19 case management, etc).  The second option refers to factors related to your organization supporting the COVID-19 response. These include a conducive atmosphere within the organization to conduct COVID-19 case management such as a strong work ethic, strong teamwork, robust coordination within the organization, robust leadership within the organization to conduct case management, accountability mechanisms within the organization, the organization’s commitment to providing support to the case management activities in terms of finance, supplies, tools, and equipment, etc. in a timely manner.  The third option refers to the simplicity of the case management devices, equipment, guidelines, tools, and manuals adapted by the organization.  The fourth option refers to the process of how case management activities were implemented, including the planning (implementation schemes/methods in advance), execution strategies (carrying out activities according to plan), engaging (attracting and involving appropriate stakeholders in implementation) reflection and evaluation of activities (monitoring of progress and quality, including regular debriefing about progress and experience), or adjustments made to the plan. |
| D402 | Among the internal contributors selected at D401, if you are asked to rank based on the magnitude of contribution, which one is the most significant contributor?  *Skip this if only one option is checked in QD401* | 1 Knowledge, skills, attitude, and self-efficacy of individuals within your organization involved in the COVID-19 case management.  2 Conducive organizational setting    3 Simplicity, feasibility, acceptability, and applicability of COVID-19 devices, equipment, guidelines, manuals  4 Well-planned and well-coordinated process of the COVID-19 case management activities | Please see the explanations above. |
| D403 | Which of the following were the external contributors to your COVID-19 case management success?  *Please check all that apply* | 1 Conducive political environment    2 Supportive economic environment  3 Conducive social environment    4 Supportive technological environment  5 Other environment (Please describe). | The first option refers to the political climate acceptance of the COVID-19 case management activities (high political will), policies and legal provisions are adequate to conduct case management, and political structure including peace and security are conducive to conducting case management.  The second option refers to the availability of sufficient revenue sources and budget to fund case management activities and/or service delivery system developments.  The third option refers to changing social norms and social support around COVID-19 case management, and conducive and accepting communities for COVID-19 case management.  The fourth option refers to technological advances outside of the organization during the time of the pandemic that could be harnessed for COVID-19 case management |
| D404 | Among the external contributors selected at D403, if you are asked to rank based on the magnitude of contribution, which one is the biggest contributor?  Skip this if only one response is checked in QD403 | 1 Conducive political environment    2 Supportive economic environment  3 Conducive social environment  4 Supportive technological environment  5 Other environment (Please describe). | Please see the explanations in question # D403 |
| D405 | Please briefly describe the most influential internal, external or combination of contributors to your program's success in completing the case management pillar | -------------------------------------------------------------------------------------------------------------------------------------------------------------------------------------------------------------------------- | Ask the respondent to describe in her/his own words the most significant contributors of the case management pillar. Please write the responses verbatim. |
| ***The following questions are about the barriers to COVID-19 case management. First, I will ask you (Question # D406) about the overall challenges related to case management. Next (Questions from D407 to D410), I will ask about the barriers related to the individuals who implemented case management including the planning, challenges related to your organization, challenges related to the case management approach itself, the processes of implementation, and the barriers external to your organization. I will ask you all these one by one.*** | | | |
| D406 | Which of the following best describes the key challenges when carrying out activities associated with COVID-19 case management?    (*Please check all that apply*) | 1 Challenges related to the knowledge, skills, attitude, and self-efficacy of individuals within your organization involved in COVID-19 case management.    2 Challenges related to your organization settings.  3 Challenges related to the complexity of the COVID-19 case management approaches and processes.  4 Challenges related to the planning, supervision, engagement, and monitoring of the case management activities.  5 Challenges related to the external settings, such us the political, technological, social and economic environment. | The first response refers to challenges related to the lack of knowledge of health workers on COVID-19 case management basics and facts. These include knowledge of how to manage cases. It also includes challenges related to the lack of self-efficacy of health workers, attitude of health workers towards COVID-19 case management, lack of motivation to conduct COVID-19 case management, resistance to change among health workers, etc.  The second option refers to challenges related to factors in the organization supporting COVID-19 case management. These include a challenging atmosphere within the organization to manage cases such as lack of structures and space, lack of internal policies, lack of work ethic in the organization, lack of teamwork, lack of quality control culture in the organization, lack of rewards and motivation policies including staff development, lack of robust coordination and leadership within the organization, lack of organization commitment to providing support to the case management activities in terms of finance, supplies, tools, and equipment, etc.  The third option refers to challenges related to the case management approach. This includes the complexity of devices, equipment, guidelines, and manuals. and lack of applicability of the technologies adapted and used by the organization. It also includes complex and non-simplistic approaches in the case management process.  The fourth option refers to challenges related to how the activity was implemented, including lack of planning, lack of execution strategies as planned, lack of reflection and evaluation of case management activities, or lack of adjustment of the plan in the changing context.  The fifth option refers to low political will, insecurity and conflict, insufficient funding or limited resources, community resistance to case management, lack of conducive technology or technology not friendly, etc. |
| D407 | What characteristic(s) best describes implementation challenges you encountered while conducting COVID-19 case management related to an individual’s characteristics?  (*Please check all that apply*)  Skip this if it is not selected in question # D406 | 1 Health personnel lack of knowledge about the activity  2 Health workers attitude and beliefs about the COVID-19 case management.  3 health personnel lack the skills to appropriately manage cases.  4 health worker personnel lack self-efficacy.  5. Health worker burnout  6 Other personal attributes (please describe) | The first option refers to health personnel who were unfamiliar with facts, truths and principles related to COVID-19 case management including diagnosis procedure, care and support, etc.  The second option refers to health personnel who did not have positive attitude towards COVID-19 case management.  The third option refers to how likely (or not) the health personnel are to provide skilled, enthusiastic, and sustained support of the COVID-19 case management throughout the different phases of pandemic.  The fourth option refers to health personnel’s lack of belief in her/his own abilities to execute required courses of action related to COVID-19 case management.  The fifth option refers to long-term stress reactions of health personnel as a result of overload, long time work, etc, and the influence of the burnout on the COVID-19 case management outcomes. |
| D408 | What characteristic(s) best describes implementation challenges to COVID-19 case management you encountered related to your organizational setting?  (*Please check all that apply)*  Skip this if it is not selected in question # C406 | 1 Lack of physical structure and space to conduct case management (for inpatient and outpatient) within the organization.  2 Lack of strong internal and external referral systems  3 Lack of internal policies, norms, and values within the organization  4 Lack of work ethic in the organization  5 Lack of teamwork  6 Lack of quality control culture in the organization    7 Lack of rewards and motivation in the organization  8 Lack of robust coordination and leadership  9 Organization lacks the commitment to providing resources for COVID-19 case management activities.  10 Other challenges attributed to the organization (please describe) | The first option refers to challenges related to physical structure which is safe both for the patient and the health personnel such as adequate and clean space for case management.  The second option refers to challenges related to lack of established internal referral systems, external referral systems, communication between and among health professionals and coordinators.  The third option refers to challenges related organization norms and values. It includes institutional culture related to use of evidence-based practice, shared mission and vision, lack of long-term experiences accumulated and recognized by the community it serves, lack of organizational readiness to adapt, relation with community it serves, rewarding policies and procedures, lack of accountability mechanisms, etc.  The eighth and ninth options refer to challenges related to lack of leadership engagement, limited available resources and poor access to knowledge and information to the case management staff. |
| D409 | What characteristic(s) best capture implementation challenges to COVID-19 case management you encountered related to COVID- 19 case management characteristics or the case management approach itself?  (*Please check all that apply*)  Skip this if it is not selected in question # D406 | 1 Lack of acceptance of the case management approach by the health workers who conduct case management.  2 The perception from the health personnel that the effectiveness of the case management approach is not supported by evidence to implement it.  3 Complexity of case management procedures, tools and approaches  4 Not well adapted to local contexts.    5 The case management approaches and activities were expensive.  6 Other (please describe) | The first option refers to the perception that the COVID-19 case management procedures and protocols are made by an external body (imposed from external bodies) and the perception that it is not appropriate for their setting.  The second option refers to the perceptions from the case management staff that the quality and validity of the evidence did not support that the management protocol would have the desired outcomes.  The third option refers to the perceived difficulty of the COVID-19 case management approach. This may include: complex devices to operate, the protocol is complex to understand, etc.  The fourth option refers to COVID-19 case management procedures and protocols were not in line with local contexts (not in line with training and readiness of health workers managing the cases, etc).  The fifth option refers to the cost of case management including devices, supply, and opportunity costs. The cost-effectiveness and the cost benefits of COVID-19 case management protocol seems inappropriate. |
| D410 | What characteristic(s) best capture implementation challenges of COVID-19 case management you encountered related to external settings?  *Please check all that apply*  Skip this if it is not selected in question # D406 | 1 Political environment  2 Economic environment    3 Social environment  4 Technological environment.  5 Other environment | The first option refers to policymaker disinterest or resistance to COVID-19 case management, limited windows of opportunity within the political climate, and political structure non-conducive to coordinated action, conflict, and unrest.  The second option refers to insufficient revenue sources to fund COVID-19 case management.  The third option refers to communities in which COVID-19 response activities were implemented that are non-accepting and/or resistant to COVID-19 case management (low health- seeking behavior for example).    The fourth option refers to slow or limited advances in technologies used in implementing COVID-19 case management.  The fifth option refers to the environment where the activity was implemented was prohibitive and did not contribute to the success of the COVID-19 pandemic response, including ineffective cross-organizational collaboration. |
| D411 | Please describe the most influential internal, external or combination of challenges you experienced when carrying out activities associated with COVID-19 case management pillar. | ---------------------------------------------------------------------------------------------------------------------------------------------------------------------------------------------------------------------------------------------------------------------------------------------------------------------------------------------------------------------------------- | Ask the respondent to describe in her/his own words the most significant challenges to the COVID-19 case management pillar. Please write the responses verbatim. |
| D412 | At what stage(s) of the process of COVID-19 response implementation did you experience the most implementation challenges related to case management?  (Please check all that apply) | 1 Early Response (March to September 2020)  2 Response (October 2020 to December 2021)  3 Mid-Response (January 2022 to September 2022)  4 Late Response (October 2022 to Present) |  |
| D413 | What were the activities you conducted to overcome these challenges? | 1)____________  2)____________  3)____________  4)____________ |  |

| E. Infection prevention and control (*Check Q204 and skip this section if the respondent was not involved in this pillar*) |
| --- |

| ***To the respondent:*** For this next set of questions, we would like to ask you about implementation facilitators and challenges relevant to the pillar of Infection prevention and control.  ***To the data collector:*** Please note any contradictions in the responses (for example between E401 (facilitator or success) and E406 (challenge)) and ask explanation from the respondent before leaving the area where the data collection is undertaken. | | | | | | | | | | | | | | |
| --- | --- | --- | --- | --- | --- | --- | --- | --- | --- | --- | --- | --- | --- | --- |
|  | Question | Response | | | | | | | | | | | Detailed explanation of each of the responses | |
| ***The next five questions ( E401 to E405) are about the facilitators or contributors of the COVID-19 Infection prevention and control.*** | | | | | | | | | | | | | | |
| E401 | Which of the following were the internal contributors to your success in the COVID-19 infection prevention and control pillar?  *Please check all that apply.* | 1 Knowledge, skills, attitude, and self-efficacy of individuals within your organization involved in the COVID-19 infection prevention and control.  2 Conducive organizational setting    3 Simplicity, applicability, and feasibility of the COVID-19 infection prevention and control approaches  4 Well-planned and well-coordinated processes of COVID-19 infection prevention and control activities | | | | | | | | The first option refers to the knowledge of staff (familiarity with facts, truths, and principles related to COVID-19 infection prevention and control), self-efficacy of staff (belief in one’s own abilities to execute required courses of action), and skills of staff (how likely or not the individual is to provide skilled, enthusiastic and sustained support of the COVID-19 infection prevention and control activities throughout the different stages of the response), attitude of health personnel towards COVID-19 infection prevention and control, motivation to conduct COVID-19 infection prevention and control, etc).  The second option refers to factors related to your organization supporting the COVID-19 response. These include a conducive atmosphere within the organization to conduct COVID-19 infection prevention and control such as strong work ethic, strong teamwork, robust coordination within the organization, robust leadership within the organization to conduct infection prevention and control, accountability mechanisms within the organization, organization’s commitment to providing support to the infection prevention and control activities in terms of finance, supplies, tools, and equipment, etc. in a timely manner.    The third option refers to the simplicity, feasibility, and applicability of infection prevention and control activities in the organization.  The fourth option refers to the process of how infection prevention and control activities were implemented, including the planning (implementation schemes/methods in advance), execution strategies (carrying out activities according to plan), engaging (attracting and involving appropriate stakeholders in implementation) reflection and evaluation of activities (monitoring of progress and quality, including regular debriefing about progress and experience), or adjustments made to the plan. | | | | |
| E402 | Among the internal contributors selected at E401, if you are asked to rank based on the magnitude of contribution, which one is the biggest contributor?  Skip this if only one response in checked in QE401 | 1 Knowledge, skills, attitude, and self-efficacy of individuals within your organization involved in the COVID-19 infection prevention and control.  2 Conducive organizational setting    3 Simplicity, feasibility, acceptability, and applicability of the COVID-19 infection prevention and control approaches  4 Well-planned and well-coordinated processes of COVID-19 infection prevention and control activities | | | | | | | | | | Please see the explanations above. | | |
| E403 | Which of the following were the external contributors to your COVID-19 infection prevention and control success?  *Please check all that apply* | 1 Conducive political environment    2 Supportive economic environment  3 Conducive social environment    4 Supportive technological environment  5 Other environment (Please describe). | | | | | | | The first option refers to the political climate accepting of the COVID-19 infection prevention and control activities (high political will), policies and legal provisions are adequate to conduct infection prevention and control, and political structure including peace and security are conducive to conducting infection prevention and control.  The second option refers to the availability of sufficient revenue sources and budget to fund infection prevention and control activities.  The third option refers to changing social norms and social support around COVID-19 infection prevention and control, and conducive and accepting communities for COVID-19 infection prevention and control.  The fourth option refers to technological advances outside of the organization during the time of the pandemic that could be harnessed for COVID-19 infection prevention and control. | | | | | |
| E404 | Among the external contributors selected at QE403, if you are asked to rank based on the magnitude of contribution, which one is the biggest contributor?  Skip this if only one response is checked in QE403 | 1 Conducive political environment    2 Supportive economic environment  3 Conducive social environment  4 Supportive technological environments  5 Other environment (Please describe). | | | | | | | Please see the explanations in question # E403 | | | | | |
| E405 | Please briefly describe the most influential internal, external or combination of contributors to your program's success in completing the infection prevention and control pillar | -------------------------------------------------------------------------------------------------------------------------------------------------------------------------------------------------------------------------- | | | | | | | | | Ask the respondent to describe in her/his own words the most significant contributors to the infection prevention and control pillar. Please write the responses verbatim. | | | |
| ***The following questions are about the barriers to COVID-19 infection prevention and control. First, I will ask you (Question # E406) about the overall challenges related to infection prevention and control. Next (Questions from E407 to E410), I will ask about the barriers related to the individuals who implemented infection prevention and control including the planning, challenges related to your organization, challenges related to the infection prevention and control approach itself, the processes of implementation and the barriers external to your organization. I will ask you all these questions one by one.*** | | | | | | | | | | | | | | |
| E406 | Which of the following best describes the key challenges when carrying out activities associated with COVID-19 infection prevention and control?    (*Please check all that apply*) | 1 Challenges related to the knowledge, skills, attitude, and self-efficacy of individuals within your organization involved in COVID-19 infection prevention and control.    2 Challenges related to your organization settings.  3 Challenges related to the complexity of COVID-19 infection prevention and control approaches and processes.  4 Challenges related to the planning, supervision, engagement, and monitoring of the infection prevention and control activities.  5 Challenges related to the external settings, such as the political, technological, social and economic environment. | | | | | The first response refers to challenges related to the lack of knowledge of individuals in the organization on COVID-19 infection prevention and control basics and facts. These include knowledge of how infection prevention and control is conducted. It also includes challenges related to the lack of self-efficacy of health workers, attitude of health workers toward COVID-19 infection prevention and control, lack of motivation to conduct COVID-19 infection prevention and control, resistance to change among individuals performing infection prevention and control, etc.  The second option refers to challenges related to factors in the organization supporting COVID-19 infection prevention and control. These include a challenging atmosphere within the organization to conduct infection prevention and control such as lack of structures and space, lack of internal policies, lack of work ethic in the organization, lack of teamwork, lack of quality control culture in the organization, lack of rewards and motivation policies including staff development, lack of robust coordination and leadership within the organization, lack of organization commitment to providing support to infection prevention and control activities in terms of finance, supplies, tools, and equipment, etc.    The third option refers to challenges related to the infection prevention and control approach. This includes the complexity of the infection prevention protocol and guidelines.  The fourth option refers to challenges related to how the activity was implemented, including lack of planning, lack of execution strategies as planned, lack of reflection and evaluation of infection prevention and control activities, or lack to adjustment of the plan in the changing context.  The fifth option refers to low political will, insecurity and conflict, insufficient funding or limited resources, community resistance to infection prevention and control, lack of conducive technology or technology not friendly, etc. | | | | | | | |
| E407 | What characteristic(s) best describes implementation challenges you encountered while conducting COVID-19 infection prevention and control related to an individual’s characteristics?  (*Please check all that apply*)  Skip this if it is not selected in question # E406 | 1 Individuals performing the task lack knowledge about the activity  2 Individuals performing the task have no positive attitude and beliefs about COVID-19 infection prevention and control.  3 Individuals performing the task lack the skills to appropriately conduct infection prevention and control.  4 health worker personnel lack self-efficacy.  5. Health worker burnout  7 Other personal attributes (please describe) | | | | | | The first option refers to personnel who were unfamiliar with facts, truths, and principles related to COVID-19 case management including diagnosis procedure, care and support, etc.  The second option refers to personnel who did not have a positive attitude toward COVID-19 infection prevention and control.  The third option refers to how likely (or not) the personnels are to provide skilled, enthusiastic, and sustained support for COVID-19 infection prevention and control throughout the different phases of the pandemic.    The fourth option refers to the personnel’s lack of belief in her/his own abilities to execute required courses of action related to COVID-19 infection prevention and control.  The fifth option refers to long-term stress reactions of personnel as a result of overload, long time work, etc, and the influence of burnout on the COVID-19 infection prevention and control outcomes. | | | | | | |
| E408 | What characteristic(s) best describes implementation challenges to COVID-19 infection prevention and control related to your organizational setting?  (*Please check all that apply)*  Skip this if it is not selected in question # E406 | 1 Lack of physical structure and space to conduct infection prevention and control appropriately within the organization.  2 Lack of internal policies, norms, and values within the organization  3 Lack of work ethic in the organization  4 Lack of teamwork  5 Lack of quality control culture in the organization    6 Lack of rewards and motivation in the organization  7 Lack of robust coordination and leadership  8 Organization lacks the commitment to providing resource for COVID-19 infection prevention and control activities.  9 Other challenges attributed to the organization (please describe) | | | | The first option refers to challenges related to physical structure space.  The second option refers to challenges related to organizational norms and values. It includes institutional culture related to the use of evidence-based practice, shared mission and vision, lack of long-term experiences accumulated and recognized by the community it serves, lack of organizational readiness to adapt, relation with the community it serves, rewarding policies and procedures, lack of accountability mechanisms, etc.  The seventh and eighth options refer to challenges related to lack of leadership engagement, limited available resources, and poor access to knowledge and information for infection prevention and control. | | | | | | | | |
| E409 | What characteristic(s) best capture implementation challenges to COVID-19 infection prevention and control you encountered related to COVID- 19 infection prevention and control characteristics?      (*Please check all that apply*)  Skip this if it is not selected in question # E406 | 1 Lack of acceptance of the infection prevention and control approaches by the personnel who conduct infection prevention and control.  2 The perception from the staff that the effectiveness of the infection prevention and control approach is not supported by evidence to implement it.    3 Complexity of infection prevention and control procedures, tools and approaches  4 Not well adapted to local contexts.    5 The infection prevention and control approaches and activities were expensive.  6 Other (please describe) | The first option refers to the perception that the COVID-19 infection prevention and control procedures and protocols are made by an external body (imposed from external bodies) and the perception that it is not appropriate for their setting.  The second option refers to the perceptions from the infection prevention and control staff that the quality and validity of the evidence did not support that the infection prevention and control protocol would have the desired outcomes.  The third option refers to the perceived difficulty of the COVID-19 infection prevention and control approach. This may include: complex infection prevention and control protocol to understand, etc.  The fourth option refers to COVID-19 infection prevention and control procedures and protocols that were not in line with local contexts.  The fifth option refers to the cost of infection prevention and control. The cost-effectiveness and the cost benefits of the COVID-19 case management protocol seem inappropriate. | | | | | | | | | | | |
| E410 | What characteristic(s) best capture implementation challenges of COVID-19 infection prevention and control you encountered related to external settings?  *Please check all that apply*  Skip this if it is not selected in question # E406 | 1 Political environment  2 Economic environment    3 Social environment  4 Technological environments.    5 Other environment | | The first option refers to policymaker disinterest or resistance to COVID-19 infection prevention and control, limited windows of opportunity within the political climate, and political structure non-conducive to coordinated action, conflict, and unrest.    The second option refers to insufficient revenue sources to fund COVID-19 infection prevention and control.  The third option refers to communities in which COVID-19 response activities were implemented that are non-accepting and/or resistant to COVID-19 infection prevention and control.    The fourth option refers to slow or limited advances of technologies used in implementing COVID-19 infection prevention and control.  The fifth option refers to the environment where the activity was implemented was prohibitive and did not contribute to the success of the COVID-19 pandemic response, including ineffective cross-organizational collaboration.. | | | | | | | | | | |
| E411 | Please describe the most influential internal, external, or combination of challenges you experienced when carrying out activities associated with COVID-19 infection prevention and control pillar. | ---------------------------------------------------------------------------------------------------------------------------------------------------------------------------------------------------------------------------------------------------------------------------------------------------------------------------------------------------------------------------------- | | | Ask the respondent to describe in her/his own words the most significant challenges to the COVID-19 infection prevention and control pillar. Please write the responses verbatim. | | | | | | | | | |
| E412 | At what stage(s) of the process of COVID-19 response implementation did you experience the most implementation challenges related to infection prevention and control?  (Please check all that apply) | 1 Early Response (March to September 2020)  2 Response (October 2020 to December 2021)  3 Mid-Response (January 2022 to September 2022)  4 Late Response (October 2022 to Present) | | |  | | | | | | | | | |
| E413 | What were the activities you conducted to overcome these challenges? | 1)____________  2)____________  3)____________  4)____________ | | | | | | | | | | | |  |

| F. Risk communication and community engagement (*Check Q204 and skip this section if the respondent was not involved in this pillar*) |
| --- |

| ***To the respondent:*** For this next set of questions, we would like to ask you about implementation facilitators and challenges relevant to the pillar of risk communication and community engagement.  ***To the data collector:*** Please note any contradictions in the responses (for example between F401 (facilitator or success) and F406 (challenge)) and ask explanation from the respondent before leaving the area where the data collection is undertaken. | | | | | | | | | | | | | | | |
| --- | --- | --- | --- | --- | --- | --- | --- | --- | --- | --- | --- | --- | --- | --- | --- |
|  | Question | Response | | | | | | | | | | | | | Detailed explanation of each of the responses |
| ***The next five questions ( F401 to F405) are about the facilitators or contributors of COVID-19 risk communication and community engagement.*** | | | | | | | | | | | | | | | |
| F401 | Which of the following were the internal contributors to your success in the COVID-19 risk communication and community engagement pillar?  *Please check all that apply.* | 1 Knowledge, skills, attitude, and self-efficacy of individuals within your organization involved in COVID-19 risk communication and community engagement.  2 Conducive organizational setting    3 Simplicity, applicability, and feasibility of the COVID-19 risk communication and community engagement approaches  4 Well-planned and well-coordinated processes of COVID-19 risk communication and community engagement activities | | | | | | | | | The first option refers to the knowledge of staff (familiarity with facts, truths, and principles related to the COVID-19 risk communication and community engagement), self-efficacy of staff (belief in one’s own abilities to execute required courses of action), and skills of staff (how likely or not the individual is to provide skilled, enthusiastic and sustained support of the COVID-19 risk communication and community engagement activities throughout the different stages of the response), the attitude of health personnel towards COVID-19 risk communication and community engagement, motivation to conduct COVID-19 risk communication and community engagement, etc).    The second option refers to factors related to your organization supporting the COVID-19 response. These include a conducive atmosphere within the organization to conduct COVID-19 risk communication and community engagement such as strong work ethic, strong teamwork, robust coordination within the organization, robust leadership within the organization to conduct risk communication and community engagement, accountability mechanisms within the organization, the organization’s commitment to providing support to the risk communication and community engagement activities in terms of finance, supplies, tools, and equipment, etc. in a timely manner.    The third option refers to the simplicity, feasibility, and applicability of the risk communication and community engagement activities in the organization.  The fourth option refers to the process of how risk communication and community engagement activities were implemented, including the planning (implementation schemes/methods in advance), execution strategies (carrying out activities according to plan), engagement (attracting and involving appropriate stakeholders in implementation) reflection and evaluation of activities (monitoring of progress and quality, including regular debriefing about progress and experience), or adjustments made to the plan. | | | | |
| F402 | Among the internal contributors selected in QF401, if you are asked to rank based on the magnitude of contribution, which one is the biggest contributor?  Skip this if only one response in QF401 is checked | 1 Knowledge, skills, attitude, and self-efficacy of individuals within your organization involved in COVID-19 risk communication and community engagement.  2 Conducive organizational setting    3 Simplicity, feasibility, acceptability, and applicability of COVID-19 risk communication and community engagement approaches.  4 Well-planned and well-coordinated processes of OVID-19 risk communication and community engagement activities | | | | | | | | | | | Please see the explanations above. | | |
| F403 | Which of the following were the external contributors to your COVID-19 risk communication and community engagement success?  *Please check all that apply* | 1 Conducive political environment    2 Supportive economic environment  3 Conducive social environment    4 Supportive technological environment  5 Other environments (Please describe). | | | | | The first option refers to the political climate accepting of the COVID-19 risk communication and community engagement activities (high political will), policies and legal provisions are adequate to conduct risk communication and community engagement, and political structure including peace and security are conducive to conduct risk communication and community engagement.    The second option refers to the availability of sufficient revenue sources and budget to fund risk communication and community engagement activities.  The third option refers to changing social norms and social support around COVID-19 risk communication and community engagement.  The fourth option refers to technological advances outside of the organization during the time of the pandemic that could be harnessed for COVID-19 risk communication and community engagement. | | | | | | | | |
| F404 | Among the external contributors selected in QF403, if you are asked to rank based on the magnitude of contribution, which one is the biggest contributor?  Skip this if only one response is checked in QF403 | 1 Conducive political environment    2 Supportive economic environment  3 Conducive social environment  4 Supportive technological environment  5 Other environment (Please describe). | | | | | | | | | | Please see the explanations in question # F403 | | | |
| F405 | Please briefly describe the most influential internal, external or combination of contributors to your program's success in completing the risk communication and community engagement pillar | -------------------------------------------------------------------------------------------------------------------------------------------------------------------------------------------------------------------------- | | | | | | | | | | | | Ask the respondent to describe in her/his own words the most significant contributors to the risk communication and community engagement pillar. Please write the responses verbatim. | |
| ***The following questions are about the barriers to COVID-19 risk communication and community engagement. First, I will ask you (Question # F406) about the overall challenges related to risk communication and community engagement. Next (Questions from F407 to F410), I will ask about the barriers related to the individuals who implemented risk communication and community engagement including the planning, challenges related to your organization, challenges related to the risk communication and community engagement approach itself, the processes of implementation and the barriers external to your organization. I will ask you all these questions one by one.*** | | | | | | | | | | | | | | | |
| F406 | Which of the following best describes the key challenges when carrying out activities associated with COVID-19 risk communication and community engagement?    (*Please check all that apply*) | 1 Challenges related to the knowledge, skills, attitude, and self-efficacy of individuals within your organization involved in COVID-19 risk communication and community engagement.    2 Challenges related to your organization settings.  3 Challenges related to the complexity of COVID-19 risk communication and community engagement approaches and processes.  4 Challenges related to the planning, supervision, engagement, and monitoring of the risk communication and community engagement activities.  5 Challenges related to the external settings, such as the political, technological, social and economic environment. | | | | The first response refers to challenges related to the lack of knowledge of individuals in the organization on COVID-19 risk communication and community engagement basics and facts. These include knowledge of how risk communication and community engagement are conducted. It also includes challenges related to lack of self-efficacy of personnel, attitude of personnel towards COVID-19 risk communication and community engagement, lack of motivation to conduct COVID-19 risk communication and community engagement, resistance to change among individuals performing risk communication and community engagement, etc.    The second option refers to challenges related to factors in the organization supporting the COVID-19 response. These include a challenging atmosphere within the organization to conduct risk communication and community engagement such as lack of structures and space, lack of internal policies, lack of work ethic in the organization, lack of teamwork, lack of quality control culture in the organization, lack of rewards and motivation policies including staff development, lack of robust coordination and leadership within the organization, lack of organization commitment to providing support to risk communication and community engagement activities in terms of finance, supplies, tools, and equipment, etc.    The third option refers to challenges related to the risk communication and community engagement approach. This includes the complexity of the risk communication and community engagement guidelines.  The fourth option refers to challenges related to how the activity was implemented, including lack of planning, lack of execution strategies as planned, lack of reflection and evaluation of risk communication and community engagement activities, or lack of adjustment of the plan in the changing context.  The fifth option refers to low political will, insecurity and conflict, insufficient funding or limited resources, lack of conducive technology or technology not friendly, etc. | | | | | | | | | |
| F407 | What characteristic(s) best describes implementation challenges you encountered while conducting COVID-19 risk communication and community engagement related to an individual’s characteristics?  (*Please check all that apply*)  Skip this if it is not selected in question # F406 | 1 Personnel performing the task lack knowledge about the activity  2 Personnel performing the task have no positive attitude and beliefs about COVID-19 risk communication and community engagement.  3 Personnel performing the task lack the skills to appropriately conduct risk communication and community engagement.  4 Personnel lack self-efficacy.  5. Staff burnout  7 Other personal attributes (please describe) | The first option refers to personnel who were unfamiliar with facts, truths, and principles related to COVID-19 risk communication and community engagement.  The second option refers to personnel who did not have a positive attitude towards COVID- risk communication and community engagement.    The third option refers to how likely (or not) the personnel are to provide skilled, enthusiastic, and sustained support of the COVID-19 risk communication and community engagement activities throughout the different phases of the pandemic.    The fourth option refers to the personnel’s lack of belief in her/his own abilities to execute required courses of action related to COVID-19 risk communication and community engagement.  The fifth option refers to long-term stress reactions of personnel as a result of overload, long time work, etc, and the influence of burnout on the COVID-19 risk communication and community engagement outcomes. | | | | | | | | | | | | |
| F408 | What characteristic(s) best describes implementation challenges to COVID-19 risk communication and community engagement related to your organizational setting?  (*Please check all that apply)*  Skip this if it is not selected in question # F406 | 1 Lack of physical structure and space to conduct risk communication and community engagement appropriately within the organization.  2 Lack of internal policies, norms, and values within the organization  3 Lack of work ethic in the organization  4 Lack of teamwork  5 Lack of quality control culture in the organization    6 Lack of rewards and motivation in the organization  7 Lack of robust coordination and leadership  8 Organization lacks the commitment to providing resource to COVID-19 risk communication and community engagement activities.  9 Other challenges attributed to the organization (please describe) | | | The first option refers to challenges related to physical structure and space.  The second option refers to challenges related organization norms and values. It includes institutional culture related to use of evidence-based practice, shared mission and vision, lack of long-term experiences accumulated and recognized by the community it serves, lack of organizational readiness to adapt, relation with community it serves, rewarding policies and procedures, lack of accountability mechanisms, etc.  The seventh and eighth options refer to challenges related to lack of leadership engagement, limited available resources and poor access to knowledge and information risk communication and community engagement. | | | | | | | | | | |
| F409 | What characteristic(s) best capture implementation challenges to COVID-19 risk communication and community engagement you encountered related to COVID-19 risk communication and community engagement characteristics?      (*Please check all that apply*)  Skip this if it is not selected in question # F406 | 1 Lack of acceptance of the risk communication and community engagement approaches by the personnel who conduct risk communication and community engagement.  2 The perception from the staff that the effectiveness of the risk communication and community engagement approach is not supported by evidence to implement it.    3 Complexity of risk communication and community engagement procedures, tools and approaches  4 Not well adapted to local contexts.    5 The risk communication and community engagement approaches and activities were expensive.  6 Other (please describe) | | The first option refers to the perception that COVID-19 risk communication and community engagement strategies and protocols are made by an external body (imposed from external bodies) and the perception that it is not appropriate for their setting.    The second option refers to the perceptions from the risk communication and community engagement staff that the quality and validity of the evidence did not support that the risk communication and community engagement strategies and protocols would have the desired outcomes.  The third option refers to the perceived difficulty of COVID-19 risk communication and community engagement approach. This may include: complex risk communication and community engagement protocol and guideline to understand, etc.    The fourth option refers to COVID-19 risk communication and community engagement procedures that were not in line with local contexts.    The fifth option refers to the cost of risk communication and community engagement. The cost effectiveness and cost-benefits of COVID-19 logistics and supply chain seems inappropriate. | | | | | | | | | | | |
| F410 | What characteristic(s) best capture implementation challenges of COVID-19 risk communication and community engagement you encountered related to external settings?  *Please check all that apply*  Skip this if it is not selected in question # F406 | 1 Political environment  2 Economic environments    3 Social environment  4 Technological environment.    5 Other environment | | | The first option refers to policymaker disinterest or resistance to COVID-19 risk communication and community engagement, limited windows of opportunity within the political climate, political structure non-conducive to coordinated action, conflict, and unrest.    The second option refers to insufficient revenue sources to fund COVID-19 risk communication and community engagement.  The third option refers to communities in which COVID-19 response activities were implemented are non-accepting and/or resistant to COVID-19 risk communication and community engagement.    The fourth option refers to slow or limited advances of technologies used in implementing COVID-19 risk communication and community engagement.  The fifth option refers to environment where activity was implemented was prohibitive and did not contribute to the success of COVID-19 pandemic response, including ineffective cross-organizational collaboration. | | | | | | | | | | |
| F411 | Please describe the most influential internal, external or combination of challenges you experienced when carrying out activities associated with COVID-19 risk communication and community engagement pillar. | ---------------------------------------------------------------------------------------------------------------------------------------------------------------------------------------------------------------------------------------------------------------------------------------------------------------------------------------------------------------------------------- | | | | | | | | Ask the respondent to describe in her/his own words the most significant challenges to COVID-19 risk communication and community engagement pillar. Please write the responses verbatim. | | | | | |
| F412 | At what stage(s) of the process of COVID-19 response implementation did you experience most implementation challenges related to risk communication and community engagement?  (Please check all that apply) | 1 Early Response (March to September 2020)  2 Response (October 2020 to December 2021)  3 Mid-Response (January 2022 to September 2022)  4 Late Response (October 2022 to Present) | | | | | | |  | | | | | | |
| F413 | What were the activities you conducted to overcome these challenges? | 1)____________  2)____________  3)____________  4)____________ | | | | | |  | | | | | | | |

| G. Logistics and supply chain (*Check Q204 and skip this section if the respondent was not involved in this pillar*) |
| --- |

| ***To the respondent:*** For this next set of questions, we would like to ask you about implementation facilitators and challenges relevant to the pillar of logistics and supply chain.  ***To the data collector:*** Please note any contradictions in the responses (for example between G401 (facilitator or success) and G406 (challenge)) and ask explanation from the respondent before leaving the area where the data collection is undertaken. | | | | | | | | | | |
| --- | --- | --- | --- | --- | --- | --- | --- | --- | --- | --- |
|  | Question | Response | | | | | | | | Detailed explanation of each of the responses |
| ***The next five questions ( G401 to G405) are about the facilitators or contributors of COVID-19 logistics and supply chain pillar.*** | | | | | | | | | | |
| G401 | Which of the following were the internal contributors to your success in COVID-19 logistics and supply chain pillar?  *Please check all that apply.* | 1 Knowledge, skills, attitude, and self-efficacy of individuals within your organization involved in COVID-19 logistics and supply chain.  2 Conducive organizational setting    3 Simplicity, applicability, and feasibility of COVID-19 logistics and supply chain approaches  4 Well-planned and well-coordinated processes of COVID-19 logistics and supply chain activities | | | | | | | The first option refers to the knowledge of staff (familiarity with facts, truths and principles related to COVID-19 logistics and supply chain), self-efficacy of staff (belief in one’s own abilities to execute required courses of action), and skills of staff (how likely or not the individual is to provide skilled, enthusiastic and sustained support of COVID-19 logistics and supply chain activities throughout the different stages of the response), attitude of personnel towards COVID-19 logistics and supply chain, motivation to conduct COVID-19 logistics and supply chain, etc).    The second option refers to factors related to your organization supporting the COVID-19 response. These include conducive atmosphere within the organization for seamless COVID-19 logistics and supply chain such as strong work ethic, strong teamwork, robust coordination within the organization, robust leadership within the organization, accountability mechanisms within the organization, the organization’s commitment to providing support to the logistics and supply chain activities in terms of finance, supplies, tools, and equipment, etc. in a timely manner.    The third option refers to the simplicity, feasibility and applicability of the logistics and supply chain activities in the organization.  The fourth option refers to the processes of how logistics and supply chain activities were implemented, including the planning (implementation schemes/methods in advance), execution strategies (carrying out activities according to plan), engaging (attracting and involving appropriate stakeholders in implementation) reflection and evaluation of activities (monitoring of progress and quality, including regular debriefing about progress and experience), or adjustments made to the plan. | |
| G402 | Among the internal contributors selected at QG401, if you are asked to rank based on the magnitude of contribution, which one is the biggest contributor?  Skip this if only one response is checked in QG401 | 1 Knowledge, skills, attitude, and self-efficacy of individuals within your organization involved in COVID-19 logistics and supply chain.  2 Conducive organizational setting    3 Simplicity, feasibility, acceptability, and applicability of COVID-19 logistics and supply chain approaches.  4 Well-planned and well-coordinated process of COVID-19 logistics and supply chain activities | | | | | | | | Please see the explanations above. |
| G403 | Which of the following were the external contributors to your COVID-19 logistics and supply chain success?  *Please check all that apply* | 1 Conducive political environment    2 Supportive economic environment    3 Supportive technological environment  5 Other environments (please describe). | | | | The first option refers to political climate acceptance of the COVI-19 logistics and supply chain activities (high political will), policies and legal provisions are adequate for COVID-19 response logistics and supply chain, and political structure including peace and security are conducive to facilitate logistics and supply chain in the area.    The second option refers to availability of sufficient revenue sources and budget to fund logistics and supply chain activities, such as purchasing logistics and supplies, establishing the logistic information system, logistic transport mechanisms, etc.  The third option refers to technological advances outside of the organization during the time of the pandemic that could be harnessed for COVID-19 logistics and supply chain. | | | | |
| G404 | Among the external contributors selected at QG403, if you are asked to rank based on the magnitude of contribution, which one is the biggest contributor?  Skip this question if only one response is checked in Qg403 | 1 Conducive political environment    2 Supportive economic environment    3 Supportive technological environment  4 Other environment (please describe). | | | | | | | | Please see the explanations in question # G403 |
| G405 | Please briefly describe the most influential internal, external or combination of contributors to your program's success in completing the logistics and supply chain pillar | -------------------------------------------------------------------------------------------------------------------------------------------------------------------------------------------------------------------------- | | | | | | | | Ask the respondent to describe in her/his own words the most significant contributors to the logistics and supply chain pillar. Please write the responses verbatim. |
| ***The following questions are about the barriers of COVID-19 logistics and supply chain pillar. First, I will ask you (Question # G406) about the overall challenges related logistics and supply chain. Next (Questions from G407 to G410), I will ask you about the barriers related to the individuals who have implemented supply chain and logistics pillar, challenges related to your organization, challenges related to the logistics and supply chain approach itself, the processes of implementation and the barriers external to your organization. I will ask you all these questions one by one.*** | | | | | | | | | | |
| G406 | Which of the following best describes the key challenges when carrying out activities associated with COVID-19 logistics and supply chain?    (*Please check all that apply*) | 1 Challenges related to the knowledge, skills, attitude, and self-efficacy of individuals within your organization involved in COVID-19 logistics and supply chain system.    2 Challenges related to your organization settings.  3 Challenges related to the complexity of COVID-19 logistics and supply chain approaches and processes.  4 Challenges related to the planning, supervision, engagement, and monitoring of the logistics and supply chain activities.  5 Challenges related to the external settings, such as the political, technological, social and economic environment. | | | | | | The first response refers to challenges related to the lack of knowledge of individuals in the organization on COVID-19 logistics and supply chain basics and facts. These include knowledge of how logistics and supply chain activities are conducted. It also includes challenges related to lack of self-efficacy of personnel, the attitude of personnel towards COVID-19 logistics and supply chain system, lack of motivation to support COVID-19 logistics and supply chain systems, resistance to change among individuals performing logistics and supply chain, etc.    The second option refers to challenges related to factors in the organization supporting COVID-19 logistics and supply chain system. These include challenging atmosphere within the organization to support logistics and supply chain such as lack of structures and space for logistics, lack of internal policies, lack of work ethic in the organization, lack of team work, lack of reward and motivation policies including staff development, lack of robust coordination and leadership within the organization, lack of organization commitment to provide support to COVID-19 logistics and supply chain activities in terms of finance, supplies, tools, and equipment, etc.    The third option refers to challenges related to the logistics and supply chain systems. This includes the complexity of the logistics and supply chain guidelines and tools, procurement mechanisms, storage transfer etc.  The fourth option refers to challenges related to how the activity was implemented, including lack of planning, lack of execution strategies as planned, lack of reflection and evaluation of logistics and supply chain activities, or lack to adjustment of the plan in the changing context.    The fifth option refers to low political will, insecurity and conflict, insufficient funding or limited resources, lack of conducive technology or technology not friendly, etc. | | |
| G407 | What characteristic(s) best describes implementation challenges you encountered while conducting COVID-19 logistics and supply chain related to an individual’s characteristics?  (*Please check all that apply*)  Skip this if it is not selected in question # G406 | 1 Individuals performing the task lack knowledge about the activity  2 Individuals performing the task have no positive attitude and beliefs about COVID-19 logistics and supply chain.  3 Individuals performing the task lack the skills to appropriately execute logistics and supply chain activities.  4 Personnel lack self-efficacy.  5. Staff burnout  7 Other personal attributes (please describe) | | | The first option refers to personnel who were unfamiliar with facts, truths and principles related to COVID-19 logistics and supply chain activities and systems.  The second option refers to personnel who did not have positive attitude towards COVID-19 logistics and supply chain system development.    The third option refers to how likely (or not) the personnel are to providing skilled, enthusiastic, and sustained support of COVID-19 logistics and supply chain activities throughout the different phases of the pandemic.    The fourth option refers to personnel’s lack of belief in her/his own abilities to execute required courses of action related to COVID-19 logistics and supply chain.  The fifth option refers to long-term stress reactions of personnel as a result of overload, long time work, etc, and the influence of burnout on the performance of COVID-19 logistics and supply chain pillar. | | | | | |
| G408 | What characteristic(s) best describes implementation challenges to COVID-19 logistics and supply chain related to your organizational setting?  (*Please check all that apply)*  Skip this if it is not selected in question # G406 | 1 Lack of physical structure and space to support/develop the logistics and supply chain system appropriately within the organization.  2 Lack of internal policies, norms, and values within the organization    3 Lack of work ethic in the organization  4 Lack of teamwork  5 Lack of quality control culture in the organization    6 Lack of rewards and motivation in the organization  7 Lack of robust coordination and leadership  8 Organization lacks commitment to providing resource to develop COVID-19 logistics and supply chain system.  9 Other challenges attributed to the organization (please describe) | | | | | The first option refers to challenges related to physical structure and space, such as space for storage that is safe and appropriate.  The second option refers to challenges related organizational norms and values. It includes institutional culture related to use of evidence-based practice, shared mission and vision, lack of long-term experiences accumulated and recognized by the community it serves, lack of organizational readiness to adapt, rewarding policies and procedures for high performing, lack of accountability mechanisms, etc.  The seventh and eighth options refer to challenges related to lack of leadership engagement, limited resources and poor access to knowledge and information for logistics and supply chain. | | | |
| G409 | What characteristic(s) best capture implementation challenges to COVID-19 logistics and supply chain you encountered related to COVID-19 logistics and supply chain characteristics?      (*Please check all that apply*)  Skip this if it is not selected in question # G406 | 1 Lack of acceptance of the logistics and supply chain approaches by the personnel who support logistics and supply chain.    2 The perception from the staff that the effectiveness of the logistics and supply chain approach adopted by the organization is not supported by evidence to implement it.    3 Complexity of the logistics and supply chain procedures, tools and approaches  4 Not well adapted to local contexts.    5 The logistics and supply chain approaches and activities were expensive.  6 Other (please describe) | | The first option refers to the perception that COVID-19 logistics and supply chain systems, procedures and activities are made by an external body (imposed from external bodies) and the perception that it is not appropriate for their setting.    The second option refers to the perceptions from the logistics and supply chain staff that the quality and validity of the evidence did not support that the logistics and supply chain system would have the desired outcomes, or the activity is just designed in ad hoc basis without proper study.  The third option refers to the perceived difficulty of COVID-19 logistics and supply chain approach. This may include: complex logistics and supply chain systems.    The fourth option refers to COVID-19 logistics and supply chain procedures that were not in line with local contexts.    The fifth option refers to the cost of logistics and supply chain. The cost effectiveness and the cost- benefits of COVID-19 logistics and supply chain seem inappropriate. | | | | | | |
| G410 | What characteristic(s) best capture implementation challenges of COVID-19 logistics and supply chain systems you encountered related to external settings?  *Please check all that apply*  Skip this if it is not selected in question # G406 | 1 Political environment  2 Economic environments    3 Technological environment    4 Other environment | The first option refers to policymaker disinterest or resistance to COVID-19 logistics and supply chain, limited windows of opportunity within the political climate, political structure non-conducive to coordinate the supply chain action, conflict, and unrest.    The second option refers to insufficient revenue sources to fund COVID-19 logistics and supply chain.    The third option refers to slow or limited advances of technologies used in implementing COVID-19 logistics and supply chain.  The fifth option refers to the environment where activity was implemented was prohibitive and did not contribute to the success of COVID-19 pandemic response, including ineffective cross-organizational collaboration. | | | | | | | |
| G411 | Please describe the most influential internal, external or combination of challenges you experienced when carrying out activities associated with COVID-19 logistics and supply chain pillar. | ---------------------------------------------------------------------------------------------------------------------------------------------------------------------------------------------------------------------------------------------------------------------------------------------------------------------------------------------------------------------------------- | | | | | | | | Ask the respondent to describe in her/his own words the most significant challenges to COVID-19 logistics and supply chain pillar. Please write the responses verbatim. |
| G412 | At what stage(s) of the process of COVID-19 response implementation did you experience most implementation challenges related to logistics and supply chain?  (Please check all that apply) | 1 Early Response (March to September 2020)  2 Response (October 2020 to December 2021)  3 Mid-Response (January 2022 to September 2022)  4 Late Response (October 2022 to Present) | | | | | | | |  |
| G413 | What were the activities you conducted to overcome these challenges? | 1)____________  2)____________  3)____________  4)____________ | | | | | | | |  |

| H. Vaccination (*Check Q204 and skip this section if the respondent was not involved in this pillar*) |
| --- |

| ***To the respondent:*** For this next set of questions, we would like to ask you about implementation facilitators and challenges relevant to the pillar of vaccination.  ***To the data collector:*** Please note any contradictions in the responses (for example between H401 (facilitator or success) and H406 (challenge)) and ask explanation from the respondent before leaving the area where the data collection is undertaken. | | | | | | | | | | | | | | | | | | | | | |
| --- | --- | --- | --- | --- | --- | --- | --- | --- | --- | --- | --- | --- | --- | --- | --- | --- | --- | --- | --- | --- | --- |
|  | | Question | | Response | | | | | | | | | | | | | | | | | Detailed explanation of each of the responses |
| ***The next five questions ( H401 to H405) are about the facilitators or contributors of COVID-19 vaccination.*** | | | | | | | | | | | | | | | | | | | | | |
| H401 | | Which of the following were the internal contributors to your success in COVID-19 vaccination pillar?  *Please check all that apply.* | | Knowledge, skills, attitude, and self-efficacy of individuals within your organization involved in COVID-19 vaccination.  2 Conducive organizational setting    3 Simplicity, applicability, and feasibility of COVID-19 vaccination approaches  4 Well-planned and well-coordinated process of COVID-19 vaccination activities | | | | | | | | | | The first option refers to the knowledge of staff (familiar with facts, truths and principles related to COVID-19 vaccines and vaccination), self-efficacy of staff (belief in one’s own abilities to execute required courses of action), and skills of staff (how likely or not the individual is to provide skilled, enthusiastic and sustained support of COVID-19 vaccination activities throughout the different stages of the response), the attitude of health personnel towards COVID-19 vaccination, motivation to conduct COVID-19 vaccination, etc).    The second option refers to factors related to your organization supporting COVID-19 response. These include a conducive atmosphere within the organization to conduct COVID-19 vaccination such as strong work ethic, strong teamwork, robust coordination within the organization, robust leadership within the organization to conduct vaccination, accountability mechanisms within the organization, the organization’s commitment to provide support to the vaccination activities in terms of finance, supplies, tools, and equipment, etc. in a timely manner.    The third option refers to the simplicity, feasibility and applicability of the vaccination approaches in the organization.  The fourth option refers to the processes of how vaccination activities were implemented, including the planning (implementation schemes/methods in advance), execution strategies (carrying out activities according to plan), engaging (attracting and involving appropriate stakeholders in implementation) reflection and evaluation of activities (monitoring of progress and quality, including regular debriefing about progress and experience), or adjustments made to the plan. | | | | | | | |
| H402 | | Among the internal contributors selected at QH401, if you are asked to rank based on the magnitude of contribution, which one is the biggest contributor?  Skip this question if only one response is checked in QH401 | | 1 Knowledge, skills, attitude, and self-efficacy of individuals within your organization involved in COVID-19 vaccination.  2 Conducive organizational setting    3 Simplicity, feasibility, acceptability, and applicability of the COVID-19 vaccination approaches  4 Well-planned and well-coordinated process of COVID-19 vaccination activities, including the planning, supervision, monitoring, and evaluation activities. | | | | | | | | | | | | | | | | | Please see the explanations above. |
| H403 | | Which of the following were the external contributors to your COVID-19 vaccination success?  *Please check all that apply* | | 1 Conducive political environment    2 Supportive economic environment  3 Conducive social environment    4 Supportive technological environment  5 Other environment (please describe). | | | | | | | | | | | | | | | The first option refers to political climate accepting of COVID-19 vaccination activities (high political will to procure and implement the program), policies and legal provisions are adequate for COVID-19 vaccination, and political structure including peace and security are conducive to conducting vaccination.  The second option refers to availability of sufficient revenue sources and budget to fund immunization activities.  The third option refers to changing social norms and social support around COVID-19 vaccination, and conducive and accepting communities for COVID-19 vaccination.  The fourth option refers to technological advances outside of the organization during the time of the pandemic that could be harnessed for COVID-19 infection prevention and control. | | |
| H404 | | Among the external contributors selected at H403, if you are asked to rank based on the magnitude of contribution, which one is the biggest contributor?  Skip this if only one response is checked in QH401 | | 1 Conducive political environment    2 Supportive economic environment  3 Conducive social environment  4 Supportive technological environment  5 Other environment (Please describe). | | | | | | | | | | | | | | | | | Please see the explanations in question # E403 |
| H405 | | Please briefly describe the most influential internal, external, or combination of contributors to your program's success in completing the vaccination pillar | | -------------------------------------------------------------------------------------------------------------------------------------------------------------------------------------------------------------------------- | | | | | | | | | | | | | | | | | Ask the respondent to describe in her/his own words the most significant contributors of the vaccination pillar. Please write the responses verbatim. |
| ***The following questions are about the barriers of COVID-19 vaccination. First, I will ask you (Question # H406) about the overall challenges related to vaccination. Next (Questions from H407 to H410), I will ask about the barriers related to the individuals who implemented vaccination, challenges related to your organization, challenges related to the vaccination approach itself, the processes of implementation and the barriers external to your organization. I will ask you all these questions one by one.*** | | | | | | | | | | | | | | | | | | | | | |
| H406 | | Which of the following best describes the key challenges when carrying out activities associated with COVID-19 vaccination?    (*Please check all that apply*) | | 1 Challenges related to the knowledge, skills, attitude, and self-efficacy of individuals within your organization involved in COVID-19 vaccination.    2 Challenges related to your organization settings.  3 Challenges related to the complexity of COVID-19 vaccination approaches and processes.  4 Challenges related to the planning, supervision, engagement, and monitoring of vaccination activities.  5 Challenges related to the external settings, such us the political, technological, social and economic environment. | | | | | | | | | | | | | | The first response refers to challenges related to lack of knowledge of individuals in the organization in COVID-19 vaccination basics and facts. These include knowledge of the vaccines and vaccination procedures. It also includes challenges related to lack of self-efficacy of health workers, attitude of health workers towards COVID-19 vaccination, lack of motivation to conduct COVID-19 vaccination, resistance to change among individual performing vaccination, etc.    The second option refers to challenges related to factors in the organization supporting COVID-19 vaccination. These include challenging atmosphere within the organization to conduct vaccination such as lack of structures and space, lack of internal policies, lack of work ethic in the organization, lack of team work, lack of quality control culture in the organization, lack of reward and motivation policies including staff development, lack of robust coordination and leadership within the organization, lack of organizational commitment to providing support to vaccination activities in terms of finance, supplies, tools, and equipment, etc.    The third option refers to challenges related to the vaccination approaches. This includes the complexity of the vaccination approaches (e.g. fixed vs outreach or mobile), guidelines, and manuals.  The fourth option refers to challenges related to how the activity was implemented, including lack of planning, lack of execution strategies as planned, lack of supervision, monitoring and evaluation of vaccination activities, or lack to adjustment of the plan in the changing context.  The fifth option refers to low political will, insecurity and conflict, insufficient funding or limited resources, community resistance for vaccination, lack of conducive technology or technology not friendly, etc. | | | |
| H407 | | What characteristic(s) best describes implementation challenges you encountered while conducting COVID-19 vaccination related to an individual’s characteristics?  (*Please check all that apply*)  Skip this if it is not selected in question # H406 | | 1 Individuals performing the task lack knowledge about the activity  2 Individuals performing the task have no positive attitude and beliefs about COVID-19 vaccination.  3 Individuals performing the task lack the skills to appropriately conduct vaccination.  4 Individuals performing the task lack self-efficacy.  5. Health worker burnout  7 Other personal attributes (please describe) | | | | | | | | | | | | The first option refers personnel who were unfamiliar with facts, truths and principles related to COVID-19 vaccination.  The second option refers to the personnel who did not have positive attitude towards COVID-19 vaccination.  The third option refers to how likely (or not) the personnel are to provide skilled, enthusiastic, and sustained support to COVID-19 vaccination throughout the different phases of pandemic.    The fourth option refers to personnel’s lack of belief in her/his own abilities to execute required courses of action related to COVID-19 vaccination.  The fifth option refers to long-term stress reactions of personnel as a result of overload, long time work, etc, and the influence of burnout on COVID-19 vaccination outcomes. | | | | | |
| H408 | | What characteristic(s) best describes implementation challenges to COVID-19 vaccination related to your organizational setting?  (*Please check all that apply)*  Skip this if it is not selected in question # H406 | | 1 Lack of physical structure and space to conduct vaccination appropriately within the organization.  2 Lack of internal policies, norms, and values within the organization  3 Lack of work ethic in the organization  4 Lack of teamwork  5 Lack of quality control culture in the organization    6 Lack of reward and motivation in the organization  7 Lack of robust coordination and leadership  8 Organization lacks commitment to provide resource to COVID-19 vaccination activities.  9 Lack of integration, and internal and external referral systems  10 Other challenges attributed to the organization (please describe) | | | | | | | | | | | | | The first option refers to challenges related to physical structure and space.  The second option refers to challenges related to organizational norms and values. It includes institutional culture related to use of evidence-based practice, shared mission and vision, lack of long-term experiences accumulated and recognized by the community it serves, lack of organizational readiness to adapt, relation with community it serves, rewarding policies and procedures, lack of accountability mechanisms, etc.  The seventh and eighth options refer to challenges related to lack of leadership engagement, limited available resources and poor access to knowledge and information for vaccination.  The ninth option refers to missed opportunities because of lack of integration of vaccination activities with other key services in health facilities, such as with inpatient and outpatient clinics. | | | | |
| H409 | | What characteristic(s) best capture implementation challenges to COVID-19 vaccination you encountered related to COVID-19 vaccination characteristics?      (*Please check all that apply*)  Skip this if it is not selected in question # H406 | | 1 Lack of acceptance of the vaccination approaches by the personnel who conduct vaccination.  2 The perception from the staff that the effectiveness of the vaccine is not supported by evidence to implement it.    3 Complexity of vaccination procedures, tools and approaches  4 Not well-adapted to local contexts.    5 The vaccination approaches and activities were expensive.  6 Other (please describe) | The first option refers to the perception that the COVID-19 vaccination approaches, procedures and protocols are made by an external body (imposed from external bodies) and the perception that it is not appropriate for their setting.  The second option refers to the perceptions from the vaccination staff that the quality and validity of the evidence did not support that the vaccines would have the desired outcomes as it is implemented without evidence.    The third option refers to the perceived difficulty of COVID-19 vaccination approach. This may include: complex vaccination approaches, difficult vaccination protocol and guideline to understand, etc.    The fourth option refers to COVID-19 vaccination procedures and protocols that were not in line with local contexts (for example designed to provide only fixed services in an area where most of the people displaced and reside in a place far from fixed site).    The fifth option refers to the cost of vaccination. The cost effectiveness and the cost-benefits of COVID-19 vaccination approaches seem inappropriate. | | | | | | | | | | | | | | | | |
| H410 | | What characteristic(s) best capture implementation challenges of COVID-19 vaccination you encountered related to external settings?    *Please check all that apply*  Skip this if it is not selected in question # H406 | | 1 Political environment  2 Economic environment    3 Social environment  4 Technological environments.    5 Other environment | | The first option refers to policymaker disinterest or resistance to COVID-19 vaccination, limited windows of opportunity within the political climate, political structure non-conducive to coordinated action, conflict, and unrest.    The second option refers to insufficient revenue sources to fund COVID-19 vaccination.  The third option refers to communities in which COVID-19 response activities were implemented are non-accepting and/or resistant to COVID-19 vaccination.    The fourth option refers to slow or limited advances of technologies used in implementing COVID-19 vaccination.    The fifth option refers to environment where activity was implemented was prohibitive and did not contribute to the success of COVID-19 pandemic response, including ineffective cross-organizational collaboration. | | | | | | | | | | | | | | | |
| H411 | | Please describe the most influential internal, external, or combination of challenges you experienced when carrying out activities associated with the COVID-19 vaccination pillar. | | ---------------------------------------------------------------------------------------------------------------------------------------------------------------------------------------------------------------------------------------------------------------------------------------------------------------------------------------------------------------------------------- | | | | | | | | | | | | | | | | | Ask the respondent to describe in her/his own words the most significant challenges of COVID-19 vaccination pillar. Please write the responses verbatim. |
| H412 | | At what stage(s) of the process of COVID-19 response implementation did you experience most implementation challenges related to vaccination?  (Please check all that apply) | | 1 Early Response (March to September 2020)  2 Response (October 2020 to December 2021)  3 Mid-Response (January 2022 to September 2022)  4 Late Response (October 2022 to Present) | | | | | | | | | | | | | | | | |  |
| H413 | | What were the activities you conducted to overcome these challenges? | | 1)____________  2)____________  3)____________  4)____________ | | | | | | | | | | | | | | | | |  |
| I. Point of entry, international travel, transport, and mass gatherings (*Check Q204 and skip this section if the respondent was not involved in this pillar*) | | | | | | | | | | | | | | | | | | | | | |
| ***To the respondent:*** For this next set of questions, we would like to ask you about implementation facilitators and challenges relevant to the pillar of point of entry, international travel, transport, and mass gatherings.  ***To the data collector:*** Please note any contradictions in the responses (for example between I401 (facilitator or success) and I406 (challenge)) and ask explanation from the respondent before leaving the area where the data collection is undertaken. | | | | | | | | | | | | | | | | | | | | | |
| ***The next five questions ( I401 to I405) are about the facilitators or contributors of the COVID-19 point of entry, international travel, transport, and mass gatherings activities in your organization.*** | | | | | | | | | | | | | | | | | | | | | |
| I401 | Which of the following were the internal contributors to your success in the COVID-19 point of entry, international travel, transport, and mass gatherings pillar?  Please check all that apply. | | 1 Knowledge, skills, attitude, and self-efficacy of individuals within your organization involved in COVID-19 point of entry, international travel, transport, and mass gatherings.  2 Conducive organizational setting    3 Simplicity, feasibility, acceptability, and applicability of COVID-19 intervention approaches at point of entry, international travel and mass gatherings  4 Well-planned and well-coordinated process of COVID-19 response activities at point of entry | | | | The firs option refers to the knowledge (familiarity with facts, truths and principles related to COVID-19 related point of entry approaches including knowledge on case definitions, case investigation procedures at point of entry, case isolation and quarantine procedures at point of entry, reporting procedures and quarantine procedures), self-efficacy (belief in one’s own abilities to execute required courses of action) of personnel at point of entry, and skills of personnel (how likely or not the individual is to provide skilled, enthusiastic and sustained support of the COVID-19 point of entry, international travel, transport, and mass gatherings activities throughout the different stages of the response), attitude of personnel towards COVID-19 interventions at point of entry, international travel, transport, and mass gatherings, etc).  The second option refers to factors related to your organization supporting COVID-19 response. These include conducive atmosphere within the organization to conduct COVID-19 interventions at point of entry and at mass gatherings such as strong work ethic, strong teamwork, robust coordination within the organization, robust leadership within the organization to conduct the activities, accountability mechanisms within the organization, the organization’s commitment to provide support to the point of entry team in terms of finance, supplies, tools, and equipment, etc. in a timely manner.  The third option refers to COVID-19 intervention at point of entry and mass gatherings itself. This includes the simplicity and feasibility of the approaches including technologies and tools used such as guidelines, manuals, and case investigation forms adapted by the organization and the simplicity, applicability, feasibility of these tools. It also includes approaches and strategies used to conduct point of entry case investigation such as isolation, quarantine, etc.  The fourth option refers to the processes of how intervention activity was implemented, including the planning (implementation schemes/methods in advance), execution strategies (carrying out the activities according to plan), engaging (attracting and involving appropriate stakeholders in implementation) monitoring and evaluation of activities (monitoring program progress and quality, including regular debriefing about progress and experience) , or adjustments made to the plan. | | | | | | | | | | | | | | |
| I402 | Among the internal contributors selected at I401, if you are asked to rank based on the magnitude of contribution, which one is the biggest contributor?  Skip this if only one response is checked in QI401 | | 1 Knowledge, skills, attitude, and self-efficacy of individuals within your organization involved in the COVID-19 point of entry, international travel, transport, and mass gatherings.  2 Conducive organizational setting    3 Simplicity, feasibility, acceptability, and applicability of the COVID-19 intervention approaches at point of entry and mass gatherings  4 Well planned and well-coordinated process of the COVID-19 response activities | | | | | | | | | | | | | | | | | Please see the explanations above. | |
| I403 | Which of the following were the external contributors to your COVID-19 intervention success at point of entry and mass gatherings?  Please check all that apply | | 1 Conducive political environment    2 Supportive economic environment  3 Conducive social environment  4 Supportive technological environment  5 Other environment (Please describe). | | | | | | The first option refers to political support, political climate accepting of COVID-19 activities (high political will) at point of entry, policies and legal provisions are adequate to conduct case investigation at point of entry and mass gatherings, and political structure including peace and security are conducive to conducting the activities at point of entry and mass gatherings.  The second option refers to availability of sufficient revenue sources and budget to fund interventions at point of entry and mass gatherings.  The third option refers to changing social norms and social support around COVID-19 interventions at point of entry and mass gatherings, and conducive and accepting communities for COVID-19 interventions at point of entry and mass gatherings.  The fourth option refers to technological advances outside of the organization during the time of the pandemic that could be harnessed for COVID-19 interventions at point of entry and mass gatherings. For example, telecommunication networks conducive to reach populations, electronic platforms are available to record and report real time data, technologies are available to support case investigation at point of entry, etc. | | | | | | | | | | | | |
| I404 | Among the external contributors selected at I403, if you are asked to rank based on the magnitude of contribution, which one is the biggest contributor? | | 1 Conducive political environment    2 Supportive economic environment  3 Conducive social environment  4 Supportive technological environment  5 Other environment (Please describe). | | | | | | | | | | | | | | | | | Please see the explanations in question # B403 | |
| I405 | Please briefly describe the most influential internal, external or combination of contributors to your program's success in completing the interventions at point of entry and mass gatherings pillar | | -------------------------------------------------------------------------------------------------------------------------------------------------------------------------------------------------------------------------- | | | | | | | | | | | | | | | | | Ask the respondent to describe in her/his own words the most significant contributors of interventions at point of entry and mass gatherings. Please write the responses verbatim. | |
| ***The following questions are about the barriers of COVID-19*** interventions at point of entry and mass gatherings***. First, I will ask you (Question # I406) about the overall challenges related to interventions at point of entry. Next (Questions from I407 to I410), I will ask about the barriers related to the individuals who implemented interventions, challenges related to your organization, challenges related to the intervention approach itself, the processes of implementation and the barriers external to your organization. I will ask you all these one by one.*** | | | | | | | | | | | | | | | | | | | | | |
| I406 | Which of the following best describes the key challenges when carrying out activities associated with COVID-19 interventions at point of entry and mass gatherings?  (Please check all that apply) | | 1 Challenges related to knowledge, skills, attitude, and self-efficacy of individuals within your organization who involved in COVID-19 interventions at point of entry and mass gatherings.    2 Challenges related to your organization settings.  3 Challenges related to the complexity, applicability and acceptability of the COVID-19 interventions at point of entry and mass gatherings.  4 Challenges related to the planning, supervision, engagement, and monitoring of the interventions at point of entry and mass gatherings.  5 Challenges related to the external settings, such us the political, technological, social and economical environment. | | | | | The first response refers to challenges related to lack of knowledge of personnel on COVID-19 basics and facts. These include knowledge of case definitions and case investigation procedures, how cases are identified, isolated, reported, and quarantined at point of entry. It also includes challenges related to lack of self-efficacy of personnel, attitude of personnel towards COVID-19 interventions at point of entry, lack of motivation to conduct COVID-19 interventions at point of entry and mass gatherings, resistance to change among personnel implementing the intervention at point of entry, etc.  The second option refers to challenges related to factors in the organization supporting COVID-19 response. These include challenging atmosphere within the organization to conducting interventions at point of entry such as lack of structures and space, lack of internal policies, lack of work ethic in the organization, lack of team work, lack of robust organizational culture in the organization, lack of use of innovation in the organization, lack of rewards and motivation policies including staff development, lack of robust coordination and leadership within the organization, lack of organization commitment to provide support to the intervention team in terms of finance, supplies, tools, and equipment, etc.  The third option refers to challenges related to the intervention design at point of entry and mass gatherings and the transport system. This includes the complexity and lack of feasibility of the intervention, including technologies and tools used such as guidelines, manuals, and case investigation forms adapted by the organization. Point of entry investigation tools lack applicability and feasibility. It also includes complex and non-simplistic approaches and strategies used to conduct point of entry case investigation such as isolation, quarantine, etc.  The fourth option refers to challenges related to how the activity was implemented, including lack of planning, lack of execution strategies as planned, lack of reflection and evaluation of activities, or lacks to adjustment of the plan in the changing context.  The fifth option refers to low political will, insecurity and conflict, insufficient funding or limited resources, resistance from the community to the activities, lack of conducive technology or technology not friendly, etc. | | | | | | | | | | | | | |
| I407 | What characteristic(s) best describes implementation challenges you encountered while conducting COVID-19 interventions at point of entry, transport and mass gatherings to an individual’s characteristics?  (Please check all that apply)  Skip this if it is not selected in question # I406 | | 1 Personnel lack of knowledge about the activity  2 Personnel attitudes and beliefs about the COVID-19 point of entry and mass gatherings activity  3 Personnel lack the skills to conduct the activity.  4 Personnel lack self-efficacy  5. Health worker burnout  7 Other personal attributes (please describe) | | | | | | | | | The first option refers to personnel who were unfamiliar with facts, truths and principles related to COVID-19 interventions at point of entry and mass gatherings including case definitions, case investigation procedures, reporting procedures, etc.  The second option refers to personnel who did not have positive attitude toward COVID-19 interventions at point of entry and mass gatherings, including the feeling that COVID-19 interventions at that point is not a priority.  The third option refers to how likely (or not) the personnel are providing skilled, enthusiastic, and sustained support of COVID-19 interventions at point of entry and mass gatherings throughout the different phases of the pandemic.  The fourth option refers to personnel’s lack of belief in her/his own abilities to execute required courses of action related to COVID-19 interventions at point of entry and mass gatherings.  The fifth option refers to long-term stress reactions of personnel as a result of overload, long time work, etc, and the influence of burnout on COVID-19 interventions at point of entry and mass gatherings. | | | | | | | | | |
| I408 | What characteristic(s) best describes implementation challenges to COVID-19 interventions at point of entry and mass gatherings you encountered related to your organizational setting?  (*Please check all that apply)*  Skip this if it is not selected in question # I406 | | 1 Lack of physical structure and space to conduct interventions at point of entry and mass gatherings within the organization.  2 Lack of internal policies, norms, and values within the organization  3 Lack of work ethic in the organization  4 Lack of teamwork  5 Lack of institutional culture in the organization    6 Lack of rewards and motivation in the organization  7 Lack of robust coordination and leadership  8 Organization lacks commitment to provide resource to COVID-19 interventions at point of entry and mass gatherings.  9 Other challenges attributed to the organization (please describe) | | | | | | | | | | The first option refers to challenges related to physical infrastructure at point of entry and organization level  The second option refers to challenges related organization norms and values. It includes institutional culture related to use of evidence-based practice, shared mission and vision, lack of long-term experiences accumulated and recognized by the community it serves, lack of organizational readiness to adapt, relation with community it serves, rewarding policies and procedures, lack of accountability mechanisms, etc.  The seventh and eighth options refer to challenges related to lack of leadership engagement, limited resources and poor access to knowledge and information to the surveillance staff. | | | | | | | | |
| I409 | What characteristic(s) best capture implementation challenges to COVID-19 interventions at point of entry and mass gatherings you encountered related to COVID- 19 interventions at point of entry and mass gatherings characteristics or the program itself?  (Please check all that apply)  Skip this if it is not selected in question # I406 | | 1 Lack of acceptance of the interventions at point of entry and mass gatherings by the people who conduct surveillance.    2 The perception from the intervention staff that the effectiveness of e approach or design is not tested systematically and lack evidence to implement it.  3 Complexity of intervention tools and approaches  4 Not well adapted to local contexts.    5 Cost of the intervention approach is expensive  6 Other (please describe) | | | | | | | The first option refers to the perception that COVID-19 intervention design is made by an external body (imposed from external bodies) and the perception that it is not appropriate for them.  The second option refers to the perception from the staff that the quality and validity of the evidence did not support that the intervention design would have the desired outcomes.  The third option refers to the perceived difficulty of COVID-19 intervention approach. This may include: case definitions are too complex to understand, complex case investigation process, time taking recording and paperwork, etc.  The fourth option refers to COVID-19 interventions at point of entry was not adapted, tailored, or refined to meet local needs.  The fifth option refers to cost of intervention related activities including intervention, supply, and opportunity costs. The cost effectiveness and the cost-benefits of the intervention approach seem inappropriate. | | | | | | | | | | | |
| I410 | What characteristic(s) best capture implementation challenges of COVID-19 intervention at point of entry you encountered related to external settings?  Please check all that apply  Skip this if it is not selected in question # I406 | | 1 Political environment  2 Economic environment    3 Social environment  4 Technological environments  5 Other environment | | | | | | | | | | | | The first option refers to policymaker disinterest or resistance to the activities, limited windows of opportunity within the political climate, political structure non-conducive to coordinated action, conflict, and unrest.  The second option refers to insufficient revenue sources to fund the activities at point of entry, mass gatherings and transport.  The third option refers to communities in which COVID-19 response activities were implemented are non-accepting and/or resistant to COVID-19 related interventions, including isolation, case investigation, quarantine, etc.    The fourth option refers to slow or limited advances of technologies used in implementing COVID-19 response activities, including communication technology and coverage of mobile and internet services, electronic recording and transfer of data, unavailability of case investigation technologies, etc.  The fifth option refers to the environment where activity was implemented was prohibitive and did not contribute to the success of COVID-19 pandemic response, including ineffective cross-organizational collaboration. | | | | | | |
| I411 | Please describe the most influential internal, external or combination of challenges you experienced when carrying out activities associated with COVID-19 interventions at point of entry, transport and mass gatherings pillar. | | ---------------------------------------------------------------------------------------------------------------------------------------------------------------------------------------------------------------------------------------------------------------------------------------------------------------------------------------------------------------------------------- | | | | | | | | Ask the respondent to describe in her/his own words the most significant challenges to the program. Please write the responses verbatim. | | | | | | | | | | |
| I412 | At what stage(s) of the process of COVID-19 response implementation did you experience the most implementation challenges related to point of entry, transport, and mass gatherings?  (Please check all that apply) | | 1 Early Response (March to September 2020)  2 Response (October 2020 to December 2021)  3 Mid-Response (January 2022 to September 2022)  4 Late Response (October 2022 to Present) | | | | | | | | | | | | | | | | |  | |
| I413 | What were the activities you conducted to overcome these challenges? | | 1)____________  2)____________  3)____________  4)____________ | | | | | | | | | | | | | | | | |  | |

| J. Maintaining essential health services during the COVID-19 pandemic (*Check Q204 and skip this section if the respondent was not involved in this pillar*) |
| --- |

| ***To the respondent:*** For this next set of questions, we would like to ask you about implementation facilitators and challenges relevant to the pillar of maintaining essential health services during the COVID-19 pandemic.  ***To the data collector:*** Please note any contradictions in the responses (for example between J401 (facilitator or success) and J406 (challenge)) and ask explanations from the respondent before leaving the area where the data collection is undertaken. | | | | |
| --- | --- | --- | --- | --- |
|  | | Question | Response | Detailed explanation of each of the responses |
| ***The next five questions ( J401 to J405) are about the facilitators or contributors of the pillar of ‘maintaining essential health services during COVID-19 pandemic’.*** | | | | |
| J401 | | Which of the following were the internal contributors to your success in maintaining essential health services during COVID-19 pandemic?  *Please check all that apply.* | Knowledge, skills, attitude, and self-efficacy of individuals within your organization  2 Conducive organizational setting    3 Well-planned and well-coordinated process of activities in the health system during the pandemic | The firs option refers to the knowledge of health providers (familiar with facts, truths and principles related to COVID-19), self-efficacy of health providers (belief in one’s own abilities to execute required courses of action), and skills of health workers, attitude of health personnel towards COVID-19, motivation of health workers, etc).  The second option refers to factors related to your organization supporting COVID-19 response. These include conducive atmosphere within the organization such as strong work ethic, strong teamwork, robust coordination within the organization, robust leadership within the organization to maintaining the performance of the health system during the pandemic, accountability mechanisms within the organization, the organization’s commitment to provide support to the key health system interventions in terms of finance, supplies, tools, and equipment, etc. in a timely manner.  The third option refers to the processes of how the activities were implemented, including the planning (implementation schemes/methods in advance), execution strategies (carrying out activities according to plan), engaging (attracting and involving appropriate stakeholders in implementation) reflection and evaluation of activities (monitoring of progress and quality, including regular debriefing about progress and experiences), or adjustments made to the plan. |
| J402 | | Among the internal contributors selected at J401, if you are asked to rank based on the magnitude of contribution, which one is the biggest contributor?  Skip this question if only one response is checked in QJ401 | 1 Knowledge, skills, attitude, and self-efficacy of individuals within your organization  2 Conducive organizational setting    4 Well-planned and well-coordinated processes and activities to maintaining the performance of the health system during the pandemic | Please see the explanations above. |
| J403 | | Which of the following were the external contributors to your success in maintaining the performance of the health system during the pandemic?  *Please check all that apply* | 1 Conducive political environment    2 Supportive economic environment  3 Conducive social environment    4 Supportive technological environment  5 Other environment (Please describe). | The first option refers to political climate accepting of COVID-19 pandemic impacts (high political will), policies and legal provisions are adequate to maintaining the performance of the health system during the pandemic, and political structure including peace and security are conducive to maintaining the performance of the health system during the pandemic.  The second option refers to availability of sufficient revenue sources and budget to fund and maintaining the performance of the health system during the pandemic.  The third option refers to changing social norms and social support in maintaining the performance of the health system during the pandemic.  The fourth option refers to technological advances outside of the organization during the time of the pandemic that could be harnessed for maintaining the performance of the health system during the pandemic. |
| J404 | | Among the external contributors selected at J403, if you are asked to rank based on the magnitude of contribution, which one is the biggest contributor?  Skip this question if only one response is checked in QJ403 | 1 Conducive political environment    2 Supportive economic environment  3 Conducive social environment  4 Supportive technological environment  5 Other environment (Please describe). | Please see the explanations in question # J403 |
| J405 | | Please briefly describe the most influential internal, external or combination of contributors to your program's success in completing ‘maintaining the performance of the health system during the pandemic pillar’ | -------------------------------------------------------------------------------------------------------------------------------------------------------------------------------------------------------------------------- | Ask the respondent to describe in her/his own words the most significant contributors of this pillar. Please write the responses verbatim. |
| ***The following questions are about the barriers to maintaining the performance of the health system during the pandemic. First, I will ask you (Question # J406) about the overall challenges related to maintaining the performance of the health system during the pandemic. Next (Questions from J407 to J410), I will ask the barriers related to the individuals who implemented the intervention, challenges related to your organization, the processes of implementation and the barriers external to your organization. I will ask you all these one by one.*** | | | | |
| J406 | | Which of the following best describes the key challenges when carrying out activities associated with maintaining the performance of the health system during the pandemic?    (*Please check all that apply*) | 1 Challenges related to the knowledge, skills, attitude, and self-efficacy of individuals within your organization involved in maintaining the performance of the health system during the pandemic.    2 Challenges related to your organization settings.  3 Challenges related to the planning, supervision, engagement, and monitoring of the activities.  4 Challenges related to the external settings, such us the political, technological, social and economic environment. | The first response refers to challenges related to lack of knowledge of health workers on basics and facts about maintaining the performance of the health system during the pandemic. These include knowledge of how to manage cases without risk of infection. It also includes challenges related to lack of self-efficacy of health workers, attitude of health workers towards COVID-19, lack of motivation of health workers, resistance to change among health workers, etc.  The second option refers to challenges related to factors in the organization. These include challenging atmosphere within the organization to maintaining the performance of the health system during the pandemic such as lack of structures and space, lack of internal policies, lack of work ethic in the organization, lack of team work, lack of quality control culture in the organization, lack of reward and motivation policies including staff development, lack of robust coordination and leadership within the organization, lack of organization commitment to provide support to health workers to maintain essential services in terms of finance, supplies, tools, and equipment, etc.  The third option refers to challenges related to how the activity was implemented, including lack of planning, lack of execution strategies as planned, lack of reflection and evaluation of the activities, or lacks to adjustment of the plan in the changing context.  The fourth option refers to low political will, insecurity and conflict, insufficient funding or limited resources, community resistance to use the facilities during the pandemic, lack of conducive technology or technology not friendly, etc. |
| J407 | | What characteristic(s) best describes implementation challenges you encountered while maintaining the performance of the health system during the pandemic related to an individual’s characteristics?  (*Please check all that apply*)  Skip this if it is not selected in question # J406 | 1 Health personnel lack of knowledge about the activity  2 Health workers attitude and beliefs on how to maintain the essential health services during pandemics.  3 health personnel lack the skills to maintain the key health interventions during the pandemic.  4 health worker lack self-efficacy.  5. Health worker burnout  7 Other personal attributes (please describe) | The first option refers to health personnel who were unfamiliar with facts, truths and principles related to maintain health system performance during pandemics.  The second option refers to health personnel who did not have positive attitude towards maintaining the performance of the health system during the pandemic.  The third option refers to how likely (or not) the health personnel are to provide skilled, enthusiastic, and sustained support to the health system throughout the different phases of pandemic.  The fourth option refers to health personnel’s lack of belief in her/his own abilities to provide and maintain the health system during the pandemic.  The fifth option refers to long-term stress reactions of health personnel as a result of overload, long time work, etc, and the influence of the burnout in maintaining the performance of the health system during the pandemic. |
| J408 | | What characteristic(s) best describes implementation challenges to maintaining the performance of the health system during the pandemic you encountered related to your organizational setting?  (*Please check all that apply)*  Skip this if it is not selected in question # J406 | 1 Lack of physical structure and space to provide essential services.  2 Lack of strong internal and external referral systems  3 Lack of internal policies, norms, and values within the organization  4 Lack of work ethic in the organization  5 Lack of teamwork  6 Lack of quality control culture in the organization    7 Lack of rewards and motivation in the organization  8 Lack of robust coordination and leadership  9 Organization lacks commitment to provide resource to maintaining the performance of the health system during the pandemic.  10 Other challenges attributed to the organization (please describe) | The first option refers to challenges related to physical structure which is safe both for the patient and the health personnel such as adequate and clean space.  The second option refers to challenges related to lack of established internal referral systems, external referral systems, communication between and among health professionals and coordinators.  The third option refers to challenges related organization norms and values. It includes institutional culture related to use of evidence-based practice, shared mission and vision, lack of long-term experiences accumulated and recognized by the community it serves, lack of organizational readiness to adapt, relation with community it serves, rewarding policies and procedures, lack of accountability mechanisms, etc.  The eighth and ninth options refer to challenges related to lack of leadership engagement, limited available resources and poor access to knowledge and information to the health workforce. |
| J409 | | What characteristic(s) best capture implementation challenges of maintaining the performance of the health system during the pandemic related to external settings?  *Please check all that apply*  Skip this if it is not selected in question # J406 | 1 Political environment  2 Economic environment    3 Social environment  4 Technological environments.  5 Other environment | The first option refers to policymaker disinterest or resistance to provide support, limited windows of opportunity within the political climate, political structure non-conducive to coordinated action, conflict, and unrest.  The second option refers to insufficient revenue sources to fund continue funding the health system during the pandemic.  The third option refers to non-accepting and/or resistant from communities to continue receiving services from health care facilities during the pandemic.    The fourth option refers to slow or limited advances of technologies used in implementing interventions related to essential services.  The fifth option refers to environment where activity implemented was prohibitive and did not contribute to the success of maintaining the performance of the health system during the pandemic, including ineffective cross-organizational collaboration. |
| J411 | | Please describe the most influential internal, external or combination of challenges you experienced when carrying out activities associated with maintaining the performance of the health system during the pandemic. | ---------------------------------------------------------------------------------------------------------------------------------------------------------------------------------------------------------------------------------------------------------------------------------------------------------------------------------------------------------------------------------- | Ask the respondent to describe in her/his own words the most significant challenges in maintaining the performance of the health system during the pandemic. Please write the responses verbatim. |
| J412 | | At what stage(s) of the process of COVID-19 response you observed the most challenges in maintaining the performance of the health system?  (Please check all that apply) | 1 Early Response (March to September 2020)  2 Response (October 2020 to December 2021)  3 Mid-Response (January 2022 to September 2022)  4 Late Response (October 2022 to Present) |  |
| I413 | What were the activities you conducted to overcome these challenges? | | 1)____________  2)____________  3)____________  4)____________ |  |
