# Supplementary material for: Turning challenges into opportunities: Lessons from Ethiopia’s COVID-19 response for strengthening health systems and health security
Source: PLOS Glob Public Health. 2025 Aug 20;5(8):e0005052. doi: 10.1371/journal.pgph.0005052 (PMC12367127; doi:10.1371/journal.pgph.0005052)
Supplement: S5 File — (DOCX) [file pgph.0005052.s005.docx]

National Assessment of COVID-19 Response in Ethiopia

**Health facility questionnaire**

| **No.** | **Question** | **Response** | | | | | **Skip to** | |
| --- | --- | --- | --- | --- | --- | --- | --- | --- |
| **1 Facility Identifiers** | | | | | | |  | |
| 100 | Facility Code | [Select code from your facility directory] | | | | |  | |
| 101 | Respondent Category  [Select all that apply] | 1 Facility director/head/CEO  2 Surveillance focal person  3 Pharmacist  4 EPI focal person  5 Laboratory personnel  96 Other (Specify)----------- | | | | |  | |
| 102 | Region | 1 Addis Ababa  2 Amhara  3 Gambella  4 Oromia  5 Somali  96 Other (specify) | | | | |  | |
| 103 | Zone (to be included while programing in ODK) |  | | | | |  | |
| 104 | Woreda (to be included while programing in ODK) |  | | | | |  | |
| 105 | Facility name |  | | | | |  | |
| 106 | Type of facility | 1 Health center  2 Primary hospital  3 General hospital  4 Specialized Hospital  96 Other ____________ | | | | |  | |
| 106a | Total Number of health care workers in the facility | _________ | | | | |  | |
| 107 | Place where the facility is found | 1 Urban  2 Rural | | | | |  | |
| 108 | Name of supervisor | [Select from the list] | | | | |  | |
| 109 | Name of data collector | [Select from the list] | | | | |  | |
| 110 | Date of data collection |  | | | | |  | |
| **Availability of inpatient services** | | | | | | | | |
| 111 | Does the facility provide inpatient services | 0 No  1 Yes | | | | | If 0, skip to  112 | |
| 111a | How many overnight/inpatient beds does the facility have in total, excluding delivery beds? | __________________ | | | | |  | |
| 111b | How many beds were reserved for COVID-19 treatment and isolation? | # for treatment________  # for isolation__________ | | | | |  | |
| **Availability of COVID-19 Services in the facility** | | | | | | |  | |
| 112 | Which COVID-19 activities have ever been implemented at this health facility?  *ENUMERATORS NOTE – PLEASE SELECT ALL THAT APPLY.*  [Branching logic to ask follow up questions only if selected. Check this and programmed ODK accordingly] | 1 COVID-19 pandemic response coordination planning and monitoring  2 Risk communication and community engagement  3 Surveillance, rapid response, and case investigation  4 Testing and laboratory services  5 Infection prevention and control  6 Case management  7 COVID-19 vaccination  8 Ensuring continuity of/maintaining essential services.  96 Other (Specify) ----------------------  98 None | | | | | If none, end interview | |
| ***The following four questions are about the overall experiences of the facility in the COVID-19 response including the successes, challenges, and lessons learned.*** | | | | | | | | |
| 113 | Please tell us your overall success, if any, in the COVID-19 response in this facility. Please list the responses.  *ENUMERATOR NOTE- PLEASE WRITE "NONE" IF THERE ARE/WERE NO SUCCESSES*  *USE COMMAS TO SEPARATE THE RESPONSES* | 1_________________________  2_________________________  3 _________________________  4_________________________ | | | | |  | |
| 114 | What were the most prominent challenges your facility faced during the COVID-19 response? Please list the responses.  *ENUMERATOR NOTE- PLEASE WRITE "NONE" IF THERE ARE/WERE NO PROMINENT CHALLENGES*  *USE COMMAS TO SEPARATE THE RESPONSES* | 1_________________________  2_________________________  3 _________________________  4_________________________ | | | | |  | |
| 115 | What lessons have been learned while responding to the COVID-19 pandemic? Please list the responses.  *ENUMERATOR NOTE- PLEASE WRITE "NONE" IF THERE ARE/WERE NO LESSONS LEARNED*  *USE COMMAS TO SEPARATE THE RESPONSES* | 1_________________________  2_________________________  3 _________________________  4_________________________ | | | | |  | |
| 116 | What mechanisms were in place to ensure the continuity or sustainment of COVID-19-related interventions in this facility? Please list the responses.  *ENUMERATOR NOTE- PLEASE WRITE "NONE" IF THERE ARE/WERE NO MECHANISMS IN PLACE*  *USE COMMAS TO SEPARATE THE RESPONSES* | 1_________________________  2_________________________  3 _________________________  4_________________________ | | | | |  | |
| ***The next questions focus on the activities in this facility on each of the COVID-19 response pillars. Please select which activities below have been implemented in your health facility. Select the duration of the activity in the context of the COVID-19 pandemic response.*** | | | | | | | | |
| Pillar 1: Coordination, planning, and monitoring (Ask this section if option 1 is checked in Q112) | | | | | | | | |
| 201 | Duration of implementation of COVID-19 national plan – health facility  *ENUMERATORS NOTE – PLEASE SELECT ALL THAT APPLY.* | 1 Yes, Early Response (March to September 2020)  2 Yes, Response (October 2020 to December 2021)  3 Yes, Mid-Response (January 2022 to September 2022)  4 Yes, Late Response (October 2022 to Present)  5 The activity has not been implemented in the facility | | | | | | If 5, skip to  203 |
| 202 | What were the successes, if any, as a result of the implementation of the national plan? Please list the responses.  *ENUMERATOR NOTE- PLEASE WRITE "NONE" IF THERE ARE/WERE NO SUCCESSES*  *PLEASE USE COMMAS TO SEPARATE THE RESPONSES* | 1_________________________  2_________________________  3 _________________________  4_________________________ | | | | | | |
| 203 | Was the facility Participated/engaged with the multi-sector coordination mechanism for COVID-19 response in the area  *ENUMERATORS NOTE – PLEASE SELECT ALL THAT APPLY.* | 1 Yes, Early Response (March to September 2020)  2 Yes, Response (October 2020 to December 2021)  3 Yes, Mid-Response (January 2022 to September 2022)  4 Yes, Late Response (October 2022 to Present)  5 No participation/engagement with the multi-sector coordination mechanism | | | | If 5, skip to  301 | | |
| 203a | Were rapid response teams (RRTs) available in the facility? | 0 No  1 Yes | | | |  | | |
| 204 | What was the role of the facility in the coordination mechanism? Please list the responses  *ENUMERATORS NOTE – PLEASE USE COMMAS TO SEPARATE THE RESPONSES* |  | | | | | | |
| 205 | What role the coordination mechanism has played in the COVID-19 pandemic response? Please list the responses.  *ENUMERATORS NOTE – PLEASE USE COMMAS TO SEPARATE THE RESPONSES* |  | | | | | | |
| **Pillar 2: Risk communication and community engagement** (Ask this section if option 2 is checked in Q112) | | | | | | | | |
| 301 | Did the facility participate during the preparation of the COVID-19 risk communication and community engagement plan  *ENUMERATORS NOTE – PLEASE SELECT ALL THAT APPLY.* | 0 not at all  1 Yes, Early Response (March to September 2020)  2 Yes, Response (October 2020 to December 2021)  3 Yes, Mid-Response (January 2022 to September 2022)  4 Yes, Late Response (October 2022 to Present)  5 Don’t know | | | | |  | |
| 301a | Has the facility developed its own risk communication and community engagement plan? | 0 No  1 Yes | | | | |  | |
| 302 | Role of the facility in risk communication and community engagement (please list the responses.  *ENUMERATORS NOTE – PLEASE USE COMMAS TO SEPARATE THE RESPONSES* |  | | | | | | |
| 303 | Was trained staff available in the facility for risk communication and community engagement | 0 No  1 Yes | | | | | | |
| 304 | Availability of tools and job aids for risk communication and community engagement.  *ENUMERATORS NOTE – Yes fully should be selected if the following are available.*   - National risk communication manual/guideline - Any job aid, including charts detailing COVID-19 mode of transmission | 0 No  1 Yes, partially  2 Yes, fully | | | | | | |
| 305 | Were resources (budget) available for risk communication and community engagement for 2015 EFY? | 0 No  1 Yes, integrated with other diseases.  2 Yes, has its own budget | | | | | | |
| 306 | Were there any mechanisms in place to capture community feedback.  *Please verify from minutes or any other documented evidence*  *ENUMERATORS NOTE – PLEASE SELECT ALL THAT APPLY.* | 1 Yes, Early Response (March to September 2020)  2 Yes, Response (October 2020 to December 2021)  3 Yes, Mid-Response (January 2022 to September 2022)  4 Yes, Late Response (October 2022 to Present)  5 No community feedback mechanism was in place | | | | | | |
| 307 | Was there an approach you thought is/was good practice in COVID-19 risk communication and community engagement in this facility you want to share with us? | 0 No ------------- Skip to 308  1 Yes | | | | | | |
| 307a | Please list the approach that is good practice in COVID-19 risk communication and community engagement in this facility.  *ENUMERATORS NOTE – PLEASE USE COMMAS TO SEPARATE THE RESPONSES* | 1. _________________________ 2. _________________________ 3. _________________________ 4. _________________________ | | | | | | |
| 308 | Were there any prominent challenges you faced to implement all activities related to risk communication and community engagement in this facility? | 0 No ------------- Skip to 310  1 Yes | | | | | | |
| 308a | What were the prominent challenges you faced to implement all activities related to risk communication and community engagement in your facility? Please list the responses.  *ENUMERATORS NOTE – PLEASE USE COMMAS TO SEPARATE THE RESPONSES* | 1_________________________  2_________________________  3_________________________  4_________________________ | | | | | | |
| 309 | Have you implemented any strategies or activities to overcome these challenges related to risk communication and community engagement? | 0 No ------------- Skip to 310  1. Yes | | | | | | |
| 309 | What were the strategy/ies and activity/ies implemented to overcome the challenges related to risk communication and community engagement? Please list your responses.  *ENUMERATORS NOTE – PLEASE USE COMMAS TO SEPARATE THE RESPONSES* | 1_________________________  2_________________________  3_________________________  4_________________________ | | | | | | |
| 310 | Is there a possibility of sustaining or continuity of the current communication and community engagement practice in the facility | 0 No-----------------------skip to 311  1 Yes  2 Don’t know--------------Skip to 401 | | | | | | |
| 310a | What mechanisms are in place to sustain current practices? | ____________________ | | | | | | |
| 311 | If the facility is unable to sustain or continue the current communication and community engagement practice, why? Please list the responses.  *ENUMERATORS NOTE – PLEASE USE COMMAS TO SEPARATE THE RESPONSES* |  | | | | | | |
| **Pillar 3: Surveillance, rapid response teams, and case investigation** (Ask this section if option 3 is checked in Q112) | | | | | | | | |
| 401 | When were COVID-19 surveillance activities implemented in the facility  *ENUMERATORS NOTE – PLEASE SELECT ALL THAT APPLY.* | 1 Early Response (March to September 2020)  2 Response (October 2020 to December 2021)  3 Mid-Response (January 2022 to September 2022)  4 Late Response (October 2022 to Present) | |  | | | | |
| 402 | Routine reporting through sentinel or non-sentinel Influenza Like Infections (ILIs), Sever Acute Respiratory Infections (SARIs), or Acute Respiratory Infections (ARIs) surveillance systems  *ENUMERATORS NOTE – PLEASE SELECT ALL THAT APPLY.* | 1 Early Response (March to September 2020)  2 Response (October 2020 to December 2021)  3 Mid-Response (January 2022 to September 2022)  4 Late Response (October 2022 to Present)  5 Routine reporting has not been implemented in the facility | | | | | | |
| 403 | Was focal person for contact tracing implementation and training available in the facility?  *ENUMERATORS NOTE – PLEASE SELECT ALL THAT APPLY.* | 1 Yes, Early Response (March to September 2020)  2 Yes, Response (October 2020 to December 2021)  3 Yes, Mid-Response (January 2022 to September 2022)  4 Yes, Late Response (October 2022 to Present)  5 Focal point for contact tracing implementation and training  have not been implemented in the facility | | | | | | |
| 403a | Was implementation of Sero epidemiological investigations or studies conducted in the facility?  *ENUMERATORS NOTE – PLEASE SELECT ALL THAT APPLY.* | 1 Yes, Early Response (March to September 2020)  2 Yes, Response (October 2020 to December 2021)  3 Yes, Mid-Response (January 2022 to September 2022)  4 Yes, Late Response (October 2022 to Present)  5 Implementation of Sero epidemiological investigations or studies have not been implemented in the facility | | | | | | |
| 404 | Was trained personnel available for surveillance? | 0 No  1 Yes | | | | | | |
| 405 | Were guidelines and tools for surveillance available in the facility?  ENUMERATORS NOTE – Yes fully should be selected if the following are available.  • National COVID-19 surveillance guideline  . Case deflations  . Case investigation forms  . Reporting forms | 0 No  1 Yes, partially  2 Yes, fully | | | | | | |
| 406 | Were resources (budget) for COVID-19 surveillance available in 2015 EFY) | 0 No  1 Yes, integrated with surveillance programs with other diseases.  2 Yes, has its own budget | | | | | | |
| 407 | Was there an approach you thought is/was good practice in COVID-19 surveillance in this facility you want to share with us? | 0 No ------------- Skip to 408  1. Yes | | | | | | |
| 407a | Please list the approach that is/was good practice in COVID-19 surveillance in this facility.  *ENUMERATORS NOTE – PLEASE USE COMMAS TO SEPARATE THE RESPONSES* | 1_________________________  2_________________________  3_________________________  4_________________________ | | | | | | |
| 408 | Were there any prominent challenges the facility faced to implement all activities related to COVID-19 surveillance in this facility? | 0 No ------------- Skip to 410  1 Yes | | | | | | |
| 408b | What were the prominent challenges you faced to implement all activities related to COVID-19 surveillance in your facility? Please list your responses.  *ENUMERATORS NOTE – PLEASE USE COMMAS TO SEPARATE THE RESPONSES* | 1_________________________  2_________________________  3_________________________  4_________________________ | | | | | | |
| 409 | Have you implemented any strategies or activities to overcome these challenges related to COVID-19 surveillance? | 0 No ------------- Skip to 410  1 Yes | | | | | | |
| 409b | What were the strategies and activities implemented to overcome the challenges related to COVID-19 surveillance? Please list your answers.  *ENUMERATORS NOTE – PLEASE USE COMMAS TO SEPARATE THE RESPONSES* | 1_________________________  2_________________________  3_________________________  4_________________________ | | | | | | |
| 410 | Was there any possibility of sustaining or continuity of the current surveillance practice in the facility? | 0 No--------------------------------skip to 411  1 Yes  2 Don’t know --------------------Skip to 501 | | | | | | |
| 410a | What mechanisms are in place to sustain the practice? | ______________ | | | | | | |
| 411 | If the facility is unable to sustain or continue the current surveillance practice, why? Please list your answers.  *ENUMERATORS NOTE – PLEASE USE COMMAS TO SEPARATE THE RESPONSES* |  | | | | | | |
| **Pillar 5: Testing and Laboratory services** (Ask this section if option 4 is checked in Q112) | | | | | | | | |
| 501 | Were testing and laboratory services implemented in the facility (either testing at the facility or specimen transport, or sending requests) | 1 Yes, Early Response (March to September 2020)  2 Yes, Response (October 2020 to December 2021)  3 Yes, Mid-Response (January 2022 to September 2022)  4 Yes, Late Response (October 2022 to Present) | | | | | | |
| 502 | Was Standards of Practice (SOP) implemented for COVID-19 Testing?  *ENUMERATORS NOTE – PLEASE SELECT ALL THAT APPLY.* | 1 Yes, Early Response (March to September 2020)  2 Yes, Response (October 2020 to December 2021)  3 Yes, Mid-Response (January 2022 to September 2022)  4 Yes, Late Response (October 2022 to Present)  5 SOP for COVID-19 Testing has not been implemented in the facility | | | | | | |
| 503 | Was there on-site testing for SARS-CoV-2 detection? | 0 No  1 Yes | | | | | | |
| 504 | Was Standards of Practice (SOP) available for COVID-19 specimen transport?  *ENUMERATORS NOTE – PLEASE SELECT ALL THAT APPLY.* | 1 Yes, Early Response (March to September 2020)  2 Yes, Response (October 2020 to December 2021)  3 Yes, Mid-Response (January 2022 to September 2022)  4 Yes, Late Response (October 2022 to Present)  5 SOP for COVID-19 specimen transport has not been implemented in the facility | | | | | | |
| 505 | On average, how quickly is your health facility able to obtain COVID-19 test results for SARS-CoV-2 detection (do not include testing processing times for antibody detection)  Average for each response time frame | ------------------------------------Hours | | | | | | |
| 506 | Was trained personnel available for laboratory testing and specimen transport | 0 No  1 Yes | | | | | | |
| 507 | Were guidelines and tools for laboratory specimen processing and testing available in the facility?  *ENUMERATORS NOTE – Yes fully should be selected if the following are available.*   - Guidelines for sample collection, and transporting, and laboratory registers are available | 0 No  1 Yes, partially  2 Yes, fully | | | | | | |
| 508 | Were resources (budget) for laboratory testing available for 2015 EFY? | 0 No  1 Yes, integrated with lab tests for other diseases.  2 Yes, has its own budget | | | | | | |
| 509 | Was there an approach you thought is/was good practice in COVID-19 laboratory testing in this facility you want to share with us? | 0 No ------------- Skip to 510  1 Yes | | | | | | |
| 509a | Please list the approach that is/was good practice in COVID-19 laboratory testing in your facility.  *ENUMERATORS NOTE – PLEASE USE COMMAS TO SEPARATE THE RESPONSES* | 1)_________________________  2)_________________________  3)_________________________  4)_________________________ | | | | | | |
| 510 | Were there any prominent challenges you faced to implement all activities related to COVID-19 lab testing in this facility? | 0 No ------------- Skip to 512  1 Yes | | | | | | |
| 510a | What were the prominent challenges you faced to implement all activities related to COVID-19 lab testing in your facility? Please list your answers.  *ENUMERATORS NOTE – PLEASE USE COMMAS TO SEPARATE THE RESPONSES* | 1_________________________  2_________________________  3_________________________  4_________________________ | | | | | | |
| 511 | Have you implemented any strategies or activities to overcome these challenges related to COVID-19 lab testing in this facility? | 0 No ------------- Skip to 512  1 Yes | | | | | | |
| 511b | What were the strategies and activities implemented to overcome the challenges related to COVID-19 lab testing in this facility? Please list your answers.  *ENUMERATORS NOTE – PLEASE USE COMMAS TO SEPARATE THE RESPONSES* | 1_________________________  2_________________________  3_________________________  4_________________________ | | | | | | |
| 512 | Is there any possibility of sustaining or continuity of the current COVID-19 lab testing practice in the facility | 0 No  1 Yes ---------------------------Skip to 601  2 Don’t know------------------Skip to 601 | | | | | | |
| 512a | What mechanisms are in place to sustain the current practice? Please list your answers. | _______________________________ | | | | | | |
| 513 | If the facility is unable to sustain or continue the current COVID-19 lab testing practice, why?  *ENUMERATORS NOTE – PLEASE USE COMMAS TO SEPARATE THE RESPONSES* |  | | | | | | |
| **Pillar 6: Infection Prevention and Control** (Ask this section if option 5 is checked in Q112) | | | | | | | | |
| 601 | Was infection prevention and control activities implemented in this facility? | 1 Yes, Early Response (March to September 2020)  2 Yes, Response (October 2020 to December 2021)  3 Yes, Mid-Response (January 2022 to September 2022)  4 Yes, Late Response (October 2022 to Present) | | | | | | |
| 602 | Do you have the following Standards of Practice in place?  *ENUMERATORS NOTE – PLEASE SELECT ALL THAT APPLY.* | Health Facility-Related IPC   1. Transmission-based precautions; 2. Decontamination of medical devices; 3. Aseptic technique of invasive procedures; 4. Specific SOPs to prevent most prevalent hospital-acquired infections; 5. Occupational health   COVID-19 Specific   1. Screening for COVID-19 (triage and inpatients); 2. Monitoring staff and inpatients for COVID-19 infections; 3. Notifications of COVID-19 infections and outbreaks for inpatients | | | | | | |
| 603 | Does the facility have the following in place?  *ENUMERATORS NOTE – PLEASE READ THE RESPONSES TO THE RESPONDENTS AND*  *SELECT ALL THAT APPLY.* | 1 There was an IPC focal person in the facility  2 The facility monitored the epidemiology of COVID-19 through the local surveillance systems  3 Triage of patients/residents with COVID-19 symptoms was in place in the facility  4 There were isolation rooms/areas readily available to be used for patients with COVID-19 symptoms  5 The facility continues to monitor personal protective equipment and critical supplies  6 Facility can reinstate all measures against COVID-19 in less than a week in the event of a new increase in local transmission  7 Has signs and posters directing patients to follow certain pathways (observe)  8 Communicates about the signs and symptoms of COVID-19  9 Communicates how to correctly perform hand hygiene, respiratory etiquette, maintain physical distance and use a mask when needed  10 WASH standards for healthcare facilities | | | | | | |
| 604 | Number of healthcare workers (HCWs) with infection prevention and control training | _______________ | | | | | | |
| 605 | Were infection prevention and control guidelines available in the facility? | 0 No  1 Yes, not observed  2 Yes, observed | | | | | | |
| 606 | Were resources (budget) for IPC activities available for 2015 EFY? | 0 No  1 Yes, for all IPC activities in the facility.  2 Yes, has budget specific to COVID-19 IPC | | | | | | |
| 607 | Was there an approach you thought is/was good practice in COVID-19 infection prevention and control in this facility you want to share with us? | 0 No ------------- Skip to 608  1 Yes | | | | | | |
| 607a | Please list the approach that is/was good practice in COVID-19 infection prevention and control in your facility.  *ENUMERATORS NOTE – PLEASE USE COMMAS TO SEPARATE THE RESPONSES* | 1)_________________________  2)_________________________  3)_________________________  4)_________________________ | | | | | | |
| 608 | Were there any challenges you faced related to COVID-19 infection prevention and control in this facility since March 2020? | 0 No ------------- Skip to 611  1 Yes | | | | | | |
| 608a | Tell us the challenges this facility has faced related to COVID-19 IPC since March 2020.  *ENUMERATORS NOTE – PLEASE SELECT ALL THAT APPLY.* | 1 Lack or frequent stockout of PPE in the facility  2 lack of infection prevention amenities in the facility (water, soap, sanitizers)  3 Lack of adherence of health workers to standard requestions  96 other (specify)_____________________________. | | | | | | |
| 609 | When did you face these challenges?  *ENUMERATORS NOTE – PLEASE SELECT ALL THAT APPLY.* | 1 Early Response (March to September 2020)  2 Response (October 2020 to December 2021)  3 Mid-Response (January 2022 to September 2022)  4 Late Response (October 2022 to Present) | | | | | | |
| 610 | Have you implemented any strategies or activities to overcome these challenges related to COVID-19 infection prevention and control in this facility? | 0 No ------------- Skip to 611  1 Yes | | | | | | |
| 610a | What were the strategies and activities implemented to overcome the challenges related to COVID-19 infection prevention and control? Could you please list the responses?  *ENUMERATORS NOTE – PLEASE USE COMMAS TO SEPARATE THE RESPONSES* | 1_________________________  2_________________________  3_________________________  4_________________________ | | | | | | |
| 611 | Possibility of sustaining or continuity of the current COVID-19 infection prevention and control practice | 0 No  1 Yes ---------------------------Skip to 701  2 Don’t know ------------------Skip to 701 | | | | | | |
| 612 | If the facility is unable to sustain or continue the practice, why? Please list your responses.  *ENUMERATORS NOTE – PLEASE USE COMMAS TO SEPARATE THE RESPONSES* |  | | | | | | |
| **Pillar 7: Case Management** (Ask this section if option 6 is checked in Q112) | | | | | | | | |
| 701 | Was COVID-19 case management services implemented in this facility?  *ENUMERATORS NOTE – PLEASE SELECT ALL THAT APPLY.* | 1 Yes, Early Response (March to September 2020)  2 Yes, Response (October 2020 to December 2021)  3 Yes, Mid-Response (January 2022 to September 2022)  4 Yes, Late Response (October 2022 to Present) | | | | | | |
| 702 | Was clinical referral pathway/systems implemented in this facility?  *ENUMERATORS NOTE – PLEASE SELECT ALL THAT APPLY.* | 1 Yes, Early Response (March to September 2020)  2 Yes, Response (October 2020 to December 2021)  3 Yes, Mid-Response (January 2022 to September 2022)  4 Yes, Late Response (October 2022 to Present)  5 No clinical referral pathway/systems have been implemented | | | | | | |
| 703 | Number of ICU beds dedicated to COVID-19 case management  *ENUMERATORS NOTE – skip this if the facility is a health center.* | __________________________ | | | | | | |
| 704 | Number of healthcare workers trained in case management | ___________________________ | | | | | | |
| 705 | Availability of Case Management guidelines in the facility (please observe) | 0 No  1, yes, not observed  2 Yes, observed | | | | | | |
| 706 | Were resources (budget) for COVID-19 case management available in the facility in the 2015 EFY? | 0 No, the budget is just the same as the pre-COVID period  1 Yes, fully integrated and the budget for inpatients and outpatients includes COVID-19 case management.  2 Yes, has budget specific to COVID-19 case management | | | | | | |
| 707 | Was there an approach or an activity you thought is/was unique or good practice in COVID-19 case management in this facility you want to share with us? | 0 No ------------- Skip to 708  1 Yes | | | | | | |
| 707a | Please list the approaches or activity that are unique or good practice in COVID-19 case management in your facility.  *ENUMERATORS NOTE – PLEASE USE COMMAS TO SEPARATE THE RESPONSES* | 1)_________________________  2)_________________________  3)_________________________  4)_________________________ | | | | | | |
| 708 | Were there any prominent challenges you faced to implement the activities related to COVID-19 case management in this facility? | 0 No ------------- Skip to710  1 Yes | | | | | | |
| 708a | What were the prominent challenges you faced to implement all activities related to COVID-19 case management in your facility? Please list your responses.  *ENUMERATORS NOTE – PLEASE USE COMMAS TO SEPARATE THE RESPONSES* | 1_________________________  2_________________________  3_________________________  4_________________________ | | | | | | |
| 709 | Have you implemented any strategies or activities to overcome these challenges related to COVID-19 case management in this facility? | 0 No ------------- Skip to 710  1 Yes | | | | | | |
| 709a | What were the strategies and activities implemented to overcome the challenges related to COVID-19 case management in this facility? Please list your answers.  *ENUMERATORS NOTE – PLEASE USE COMMAS TO SEPARATE THE RESPONSES* | 1_________________________  2_________________________  3_________________________  4_________________________ | | | | | | |
| 710 | Possibility of sustaining or continuity of the current COVID-19 case management practice in the facility | 0 No  1 Yes---------------------------Skip to 801  2 Don’t know-----------------Skip to 801 | | | | | | |
| 711 | If the facility is unable to sustain or continue the practice, why? Please list your answers.  *ENUMERATORS NOTE – PLEASE USE COMMAS TO SEPARATE THE RESPONSES* |  | | | | | | |
| **Pillar 8: Maintaining essential health services and systems** (Ask this section if option 8 is checked in Q112) | | | | | | | | |
| 801 | Was there any disruption or impact on the essential health services in this facility because of the COVID-19 pandemic?  *ENUMERATORS NOTE – PLEASE SELECT ALL THAT APPLY.* | 1 Yes, Early Response (March to September 2020)  2 Yes, Response (October 2020 to December 2021)  3 Yes, Mid-Response (January 2022 to September 2022)  4 Yes, Late Response (October 2022 to Present)  5 No disruption or impact on essential health services happened because of the COVID-19 pandemic | | | | | If 5, skip to 805 | |
| 802 | Which essential services were affected because of the pandemic?  *ENUMERATORS NOTE – PLEASE SELECT ALL THAT APPLY.* | 1 Childhood immunization services  2 ANC services  3 Delivery services  4 Post-natal services  5 Family planning services  6 Outpatient services  7 Inpatient services  96 Other services __________________________ | | | | | | |
| 803 | At which phase of the pandemic the disruption was most prominent? | 1 Early Response (March to September 2020)  2 Response (October 2020 to December 2021)  3 Mid-Response (January 2022 to September 2022)  4 Late Response (October 2022 to Present) | | | | | | |
| 804 | What were the most common reasons for the disruption?  *ENUMERATORS NOTE – PLEASE SELECT ALL THAT APPLY.* | 1 Shift in resources for COVID-19 pandemic response  2 Lack of adequate health workers as the workforce moved to the pandemic response.  3 Supply chain disruptions  4 Health worker panic and avoid providing good quality service because of fear of infections.  5 Clients avoid health facilities because of fear of infections.  96 Others, Specify ____________________ | | | | | | |
| 805 | Has this facility ever implemented any interventions or activities to prevent or reverse the disruption of essential health services?  *ENUMERATORS NOTE – PLEASE SELECT ALL THAT APPLY.* | 1 Yes, Early Response (March to September 2020)  2 Yes, Response (October 2020 to December 2021)  3 Yes, Mid-Response (January 2022 to September 2022)  4 Yes, Late Response (October 2022 to Present)  5 No interventions or activities implemented to prevent or reverse the disruption of essential health services | | | If 5, skip to  901 | | | |
| 806 | What interventions were implemented to reverse or prevent the disruption of essential health services in your facility?  *ENUMERATORS NOTE – PLEASE SELECT ALL THAT APPLY.* | 1 Deploying additional health care workers.  2 Installing infection prevention and control amenities to prevent new infections within the facility.  3 Installing infection prevention amenities to prevent health workers’ infection in the facility.  4 Communication and community engagement to increase communities’ confidence in the health facility.  5 Mobilizing additional resources from the government and other sources to finance health programs.  96 Others, specify ___________________ | | | | | | |
| 807 | Was there an approach you thought is/was good practice in maintaining essential health services in your facility you want to share with us? | 0 No ------------- Skip to 808  1 Yes | | | | | | |
| 807a | Please list the approachs implemented in the facility that are good practice in maintaining essential health services in your facility.  *ENUMERATORS NOTE – PLEASE USE COMMAS TO SEPARATE THE RESPONSES* | 1)_________________________  2)_________________________  3)_________________________  4)_________________________ | | | | | | |
| 808 | What do you recommend to prevent disruption of essential services in future outbreaks and pandemics?  *ENUMERATORS NOTE – PLEASE USE COMMAS TO SEPARATE THE RESPONSES* |  | | | | | | |
| 809 | Were there new developments or improvements in the healthcare system just because of the COVID-19 pandemic? | 0 No------------------------------Skip to 901  1 Yes  2 Don’t know-------------------Skip to 901 | | | | | | |
| 810 | What were the new developments in the healthcare system after the pandemic has occurred in Ethiopia? | 1 Continuous medical education practiced by many healthcare providers as Telemedicine expanded during the pandemic.  2 New devices and equipment imported/donated to the facility because of the COVID-19 pandemic and these devices and equipment improved service delivery in the facility.  3 The quality improvement tools introduced to the facility as a result of the COVID-19 pandemic improved the quality and safety of service delivery in the facility.  4 Improved supplies and equipment to the facility (e.g.: surgical masks, PPE, Sanitizers, etc)  96 Other, specify__________________________ | | | | | | |
| 811 | Do you think that these developments will sustain after the pandemic is over? | 0 No  1 Yes  2 Don’t know | | | | | | |
| **Pillar 10: COVID-19 Vaccine distribution, implementation and coverage** (Ask this section if option 7 is checked in Q112) | | | | | | | | |
| 901 | Have you been delivering COVID-19 immunization services in this facility? | 0 No  1 Yes | If 0, skip to 1000 | | | | | |
| 902 | Which immunization approaches this facility has used to reach all the target populations?  Select all that apply | 1 Fixed services  2 Outreach  3 Mobile  4 Campaign mode – Temporary site for COVID-19 mass vaccination.  5 School-based mass vaccination  96 Other (specify)-------------- | If 1, 4, 5, 6, skip to 904 | | | | | |
| 903 | How many mobile teams and outreach sites did you host in the COVID-19 immunization program? | # of outreach sites _________  # of mobile teams__________ | | | | | | |
| 904 | Which immunization approaches the facility has used to reach underserved and special populations?  *ENUMERATORS NOTE – PLEASE SELECT ALL THAT APPLY.* | 1 Fixed services  2 Outreach  3 Mobile  4 Campaign mode – Temporary site for COVID-19 mass vaccination.  5 School-based mass vaccination  96 Other (specify)--------------  98 No special population in the catchment | | | | | | |
| 905 | Have you collaborated with community- or faith-based organizations during outreach or mobile immunization programs? | 0 No  1 Yes | If 0, skip to 908 | | | | | |
| 906 | Which community and faith-based organizations have you worked with?  *ENUMERATORS NOTE – PLEASE USE COMMAS TO SEPARATE THE RESPONSES* | Name of Organizations:  1__________________  2___________________  3__________________  4___________________  5___________________ |  | | | | | |
| 907 | What was the added value of working with these organizations? Could you please list them?  *ENUMERATORS NOTE – PLEASE USE COMMAS TO SEPARATE THE RESPONSES* | 1__________________  2___________________  3__________________  4___________________ |  | | | | | |
| 908 | Has the COVID-19 immunization program already been integrated with the existing EPI program? | 0 No  1 Yes, (with fixed vaccination services)  2 Yes, (with PIRI)  3 Yes, (outreach sites/mobile teams)  4 Yes, with all approaches |  | | | | | |
| 909 | Has the COVID-19 immunization program integrated with other health services in the facility? | 0 No  1 Yes | If 0, skip to 911 | | | | | |
| 910 | With which services the immunization program is integrated in the facility? | 1 Outpatient department  2 Impatient services  3 HIV/ART clinics  4 TB clinics  5 Mass drug administration for NTDs (e.g Trachoma)  6 Insecticide-treated nets distribution.  96 Other, specify |  | | | | | |
| 911 | Have you ever encountered a stockout of any of the vaccines over the last three months? | 0 No  1 Yes |  | | | | | |
| 912 | Was there an approach you thought is/was good practice in delivering COVID-19 vaccination services in your facility you want to share with us? | 0 No ------------- Skip to 913  1 Yes | | | | | | |
| 912 | Please list the approach or activity that is unique or good practice in delivering COVID-19 vaccination services in your facility.  *ENUMERATORS NOTE – PLEASE USE COMMAS TO SEPARATE THE RESPONSES* | 1)_________________________  2)_________________________  3)_________________________  4)_________________________ | | | | | | |
| 913 | Were there any prominent challenges you faced in implementing the COVID-19 vaccination program in your facility? | 0 No ------------- Skip to 914  1 Yes |  | | | | | |
| 913a | What were the prominent challenges you experienced in implementing the COVID-19 vaccination program in your facility?  *ENUMERATORS NOTE – PLEASE SELECT ALL THAT APPLY.* | 1 Vaccine hesitancy  2 Mis and disinformation about vaccines  3 lack of vaccine supply and/or stockout  4 Difficulty in maintaining vaccine cold-chain  5 Provider knowledge and skills  6 Providers' attitude towards COVID-19 vaccines  7 Lack of resources (finance for campaigns, lack of transport for outreach and mobile teams, etc)  8 Instability, unrest, and insecurity  96 Other, Specify _________ |  | | | | | |
| 914 | What do you recommend for improving COVID-19 immunization coverage in your area?  *ENUMERATORS NOTE – PLEASE USE COMMAS TO SEPARATE THE RESPONSES* |  |  | | | | | |

| FOR ENUMERATOR | | | |
| --- | --- | --- | --- |
| 1000 | Geographic coordinate (facility location)  PLEASE RECORD THE COORDINATE IN THE COMPOUND OF THE FACILITY IN FRONT OF THE MAIN GATE |  |  |
| 1001 | Remark/General Observation, if any. | ______________________________________________________________________________________________________ |  |
