# Supplementary material for: Turning challenges into opportunities: Lessons from Ethiopia’s COVID-19 response for strengthening health systems and health security
Source: PLOS Glob Public Health. 2025 Aug 20;5(8):e0005052. doi: 10.1371/journal.pgph.0005052 (PMC12367127; doi:10.1371/journal.pgph.0005052)
Supplement: S6 File — (DOCX) [file pgph.0005052.s006.docx]

**National Assessment on COVID-19 response in Ethiopia: Experiences, Lessons Learned and Future Directions**

**Guiding Questions for Key Informant Interviews**

**Target Population**: Policy Makers, Programmers, and Implementers of the COVID-19 Response in Ethiopia

Thank you for meeting with me today.

We're interested in learning about your experiences with the COVID-19 pandemic response activities that you have led or been a part of. Specifically, we want to know about what occurred, what worked, what didn’t work, the challenges you faced while doing this work, and how you were able to tackle these challenges, as well as what you learned and think others could learn.

The questions I am going to ask don't have right or wrong answers. This conversation is completely confidential, and we can skip any question that you prefer not to answer.

Is it okay if I audio record our conversation today, for the purposes of transcription and documentation? It will not be shared with anyone outside the research team.

I. Demographics

| Respondent ID (*Should be the same us the name of the audio file*) |  |
| --- | --- |
| Age in years | _________years |
| Gender | 1 Male  2 Female |
| Organization |  |
| Position while working as COVID-19 response team |  |
| Current position |  |
| Total years of experience working |  |

**2. General information about the COVID-19 pandemic response**

| 2.1. What kind of work have you done with the COVID-19 pandemic response?  Check all that apply | 1 Legislation and policy  2 Coordination, risk communication, community engagement and advocacy  3 Infection prevention and control  4 COVID-19 vaccination  5 COVID-19 laboratory systems and testing  6 Surveillance  7 Point of entry  8 Case management  9 COVID-19 related data collection, analysis and reporting |
| --- | --- |
| 2.2. Tell me a bit more about the COVID-19 pandemic response activities in Ethiopia you were involved. | |

1. **Legislation and policy (***Ask this if 2.1 = 1***)**
   1. Which legislations have been established to prevent and control the COVID-19 pandemic in Ethiopia? When we say legislation, we mean proclamations and laws established by the parliament, council of ministers and regional councils.
   2. How did these legislations support implementation of the COVID-19 pandemic response strategies and activities?
   3. What were the adverse impacts of those legislations?
   4. What were the successes because of the implementation of those legislations?
   5. What policies and strategies were established to contain the COVID-19 pandemic?
   6. What were the challenges to implement those policies and strategies?

*Prob: Where did the challenges originate (personal, organizational, political, social, technological)?*

*Prob: How did you or your team deal with these challenges?*

- 1. What were the mechanisms in place to adapt policies and strategies in the constantly changing contexts?
  2. What lessons were learned during and after the establishment of such policies and strategies?

**4. Coordination, risk communication, community engagement and advocacy (***Ask this if 2.1 = 2***)**

- 1. When did multi-sectoral, multistakeholder coordination mechanism for COVID-19 preparedness and response established, if any, in Ethiopia/in your region/in your woreda? Was that a stand-alone coordination mechanism or part of the wider emergency coordination mechanism?
  2. How effective was the multi-sectoral, multi-stakeholder coordination mechanism in the prevention and control of the COVID-19 pandemic? If the coordination mechanism was not effective, why?
  3. What mechanisms were in place to facilitate coordination between neighboring countries?
  4. Within which sectors social distancing policies were implemented? To what extent sectors, such as transportation sectors complied with social distancing policies?
  5. To what extent have rumor management mechanisms been in place and operational?
  6. What approaches were used to raise awareness about COVID-19, to improve knowledge about COVID-19 and to increase the desired practices (e.g., door-to-door outreach, public meetings, health fairs, print/radio, social media, etc).
  7. Which of the approaches were most effective and why? What were the challenges and strengths of each of these approaches?
  8. What challenges were faced while implementing the risk communication and community engagement pillar?

*Prob: Where did the challenges originate (personal, organizational, political, social, technological)?*

*Prob: How did you or your team deal with these challenges?*

1. **Infection prevention and control (***Ask this if 2.1 = 3***)**
   1. What infection prevention interventions and mechanisms were implemented at various phases of the pandemic?
   2. What were the strengths of each infection prevention approach? What were the successes?
   3. How do infection prevention supplies were budgeted, financed, distributed, and used? What was the source of the budget? What was the contribution of the government in financing infection prevention supplies?
   4. How effective was the supply chain system to ensure availability of infection prevention supplies?
   5. To what extent the COVID-19 infection prevention activities integrated into the existing infection prevention activities at health care facilities?
   6. Tell us the challenges you encountered related to COVID-19 infection prevention and control?

*Probe: Political barriers, organizational barriers, individual barriers, social barriers, economic barriers*

- 1. What were the strategies you and your team developed to address these challenges?

Probe: who was involved in developing these strategies?

Did it work?

Probe: How do you know it worked or didn’t work? Did they bring about any positive or negative change?

How did people react to these changes?

Probe: e.g. Resistance? Acceptance?

Did it result in any changes based on these solutions?

Probe: e.g. change in protocol or operational procedures

What would have made it easier to tackle this challenge?

Probe: more people, a better COVID-19 infection prevention supply strategies system to avoid stockouts, more responsive leaders in the health care system, etc.

- 1. How differently could infection prevention policies and approaches be implemented to bring maximum results?

1. **Vaccination (***Ask this if 2.1 = 4***)**
   1. What vaccination modalities have been followed to reach target populations with COVID-19 vaccines in Ethiopia?
   2. How inclusive is the current COVID-19 vaccination program? What strategies are used to reach underserved and/or special populations?
   3. To what extent the vaccination program has achieved its targets? Please provide examples.
   4. What were the good practices related to COVID-19 vaccination, if any?
   5. What were the drivers for reaching the target populations?
   6. What were the bottlenecks/challenges to reach the target population using the current vaccination modalities?

*Probe: Political barriers, organizational barriers, individual barriers, social barriers, economic barriers, technological barriers*

- 1. What were the strategies you and your team developed to address these challenges?

Probe: who was involved in developing these strategies?

Did it work?

Probe: How do you know it worked or didn’t work? Did they bring about any positive or negative change?

How did people react to these changes?

Probe: e.g. Resistance? Acceptance?

Did it result in any changes based on these solutions?

Probe: e.g. change in protocol or operational procedures

What would have made it easier to tackle this challenge?

Probe: more people, a better COVID-19 vaccine supply chain system to avoid stockouts, more responsive leaders in the health care system, community engagement? etc.

- 1. What was the contribution of the government in financing COVID-19 vaccination programs? What is the prospect of increasing funding from the government for vaccination?
  2. What lessons could be learned from the current COVID-19 vaccination approaches?
  3. How sustainable is the current vaccination modalities? To what extent the vaccination program is integrated to existing EPI program to date?

1. **National and sub-national laboratory systems (***Ask this if 2.1 = 5***)**
   1. What was the level of the Coronavirus disease laboratory capacity in the country? How accessible were the laboratories? Were there population groups who have no access to COVID-19 laboratories? Who was excluded or did not have access to the laboratory service and why?

*Prob: IDPs, refugees, people in remote areas. Please describe the changes in the COVID-19 laboratory services overtime since the first COVID-19 case was reported in Ethiopia?*

- 1. What mechanisms were in place to ensure availability of test-kits? How effective was the supply chain system to insure uninterrupted availability of test kits and other laboratory supplies?
  2. What were the challenges to ensure availability of good quality COVID-19 laboratories?
  3. What were the strategies implemented to overcome those challenges?
  4. To what extent the COVID-19 laboratory has been integrated into the existing laboratory in the health system?
  5. How and to what extent external and internal quality control has been undertaken and how frequently quality control is undertaken?
  6. What were the mechanisms in place to ensure sustained availability of COVID-19 laboratory services?
  7. What were the laboratory related good practices that should be documented and scaled up in Ethiopia?
  8. What lessons could be learned to improve laboratory services and better prepare for future similar pandemics?

1. **Surveillance (***Ask this if 2.1 =6* **)**
   1. What mechanisms were in place to detect COVID-19 cases as early as possible? To what extent the COVID-19 surveillance system is integrated to the existing surveillance system?
   2. How do surveillance data were collected and analyzed? Comment on the timeliness, completeness and usability of surveillance data.
   3. How often decisions were made based on surveillance data?
   4. How effective is the current surveillance system? Please, provide examples that shows its effectiveness.
   5. What are the drawbacks and barriers of the current COVID-19 surveillance system?
   6. Where do the sources of those drawbacks and barriers originate?

*Probe: Political barriers, organizational barriers, individual barriers, social barriers, economic barriers*

- 1. What were the strategies you and your team developed to address these challenges?

*Probe: Were those strategies working? How do you know it is working? Please give examples.*

- 1. What contact tracing mechanisms were in place?
  2. How differently the COVID-19 surveillance be implemented to improve case identification as early as possible?
  3. What lessons were learned from the COVID-19 surveillance system in Ethiopia?
  4. How adequate is the current surveillance system to detect future outbreaks and epidemics?

1. **Point of entry (***Ask this if 2.1 = 7***)**
   1. Where and when was point of entry facilities established? What other diseases were screened at point of entry?
   2. To what extent point of entry facilities were equipped and staffed? How adequate was the preparation at the point of entry?
   3. What were the challenges faced at the point of entry facilities? What were the activities undertaken to overcome the challenges?
   4. To what extent the COVID-19 related activities at point of entry was integrated to the activities implemented for other diseases?
   5. What were the success and good practices at the point of entry?
2. **Case management and facility readiness (***Ask this if 2.1 = 8***)**
   1. To what extent case management centers were accessible to all populations? What mechanisms were in place to reach special populations, such as IDPs and populations in remote areas?
   2. To what extent case management centers were equipped and staffed with trained personnel?
   3. What quality improvement mechanisms were in place at case management centers?
   4. What were the challenges to provide good quality services at case management centers?
   5. What were the mechanisms implemented to overcome the challenges?

*Probe: Were those strategies working? How do you know it is working? Please give examples.*

- 1. What strategies were used to expand and equipe intensive care units?
  2. What lessons were learned to expand intensive care units for other health problems?
  3. To what extent case management centers were integrated to the existing health care system?

1. **COVID-19 Planning, Monitoring and Evaluation (***Ask this if 2.1 = 9***)**
   1. How was planning for COVID-19 response done? Who were the stakeholders that were engaged during planning?
   2. How was the COVID-19 data collected, analyzed and used?
   3. To what extent the COVID-19 data reporting system was integrated to the existing routine health information systems?
   4. How do day to day monitoring of the COVID-pandemic was done?
   5. To what extent staff were deployed to perform all COVID-19 related tasks, including program monitoring?
   6. What were the challenges in COVID-19 data collection, collation, and reporting?
   7. What unique approaches were in place to have good quality data?
   8. Tell me the good practices and success in terms of COVID-19 data collection, collation, analysis and reporting?
   9. What lessons are learned from COVID-19 data collection, analysis and reporting systems?
2. **Integration with the existing health system (***Ask this to all study participants***)**
   1. Overall, what was your assessment on the level of integration of the COVID-19 response program to the broader health system?
   2. What lessons were learned from the COVID-19 response integration with the broader health system?
   3. What lessons were learned in preparing for future outbreaks and pandemics?
3. **Good practices, and prospects for future prevention (***Ask this to all study participants***)**
   1. Overall, what were the good practices during the COVID-19 response in Ethiopia?
   2. How do Ethiopia use these good practices to develop resilient health systems and prevent future emergencies?
4. **Maintaining Essential Health Services and Systems (***this question should be for all study participants)*
   1. To what extent the health system was disrupted because of the COVID-19 pandemic?
   2. Which services were most affected by the pandemic?
   3. What were the reasons for the disruptions?
   4. What actions were taken to prevent health system disruptions?
   5. What were the outcomes of those actions?
   6. What lessons were learned from health systems disruptions and subsequent actions?
5. **Financing the COVID-19 Pandemic Response**
   1. To what extent the Government of Ethiopia is financing the COVID-19 response? Who else is funding the COVID-19 response? Were there plans that remained without funds? Which plans remained without funds?
   2. How effectively did the program use its funds?
6. **Lessons Learned (***All study participants should be asked this question***)**

Generally, translating the knowledge learned from the COVID-19 pandemic response efforts in Ethiopia is one of the objectives of this study. The lessons learned during the COVID-19 pandemic response may be used in preventing and responding to future pandemics.

- 1. Generally, what did you learn from the COVID-19 pandemic response?
  2. How have assets and/or contributions *(for example s*kills, tools, infrastructure, people etc.) from the COVID-19 pandemic response been useful for other health programs, such as in developing resilient health systems?
  3. Can you share any other lessons that you have learned from your work with the COVID-19 response that you think others should know?

*How have you used any of these lessons in other areas of your work and in improving public health emergency management?*

- 1. How else do you think lessons from the COVID-19 pandemic response activities would be used in other areas of health? In other sectors beyond health?
  - Do you think there were any missed opportunities for applying lessons from the COVID-19 response to other health interventions?

1. **Recommendations for effective control (***All study participants should be asked this question***)**

15.1. What do you recommend to improving the emergency preparedness and response activities in Ethiopia? *For each recommendation ask*:

- How will this action improve the country’s preparedness for health emergencies?
- What does implementation of this recommendation take? How feasible and applicable is the recommended intervention or approach?
- Who should be responsible to implement the recommendation?
