# Supplementary material for: Turning challenges into opportunities: Lessons from Ethiopia’s COVID-19 response for strengthening health systems and health security
Source: PLOS Glob Public Health. 2025 Aug 20;5(8):e0005052. doi: 10.1371/journal.pgph.0005052 (PMC12367127; doi:10.1371/journal.pgph.0005052)
Supplement: Attachment — Submitted filename: Response to Reviewers.docx [file pgph.0005052.s009.docx]

Dear Reviewers,

Thank you for reviewing our manuscript, "Turning Challenges into Opportunities: Lessons from Ethiopia’s COVID-19 Response for Strengthening Health Systems and Health Security" (Manuscript ID: PGPH-D-25-00504). We appreciate your valuable feedback, which, along with the editor's comments, has significantly strengthened our manuscript. We have thoroughly addressed all the comments and questions raised. Our detailed point-by-point responses are provided below.

Sincerely,

Muluneh Yigzaw Mossie

On behalf of the co-authors: Jenny Shannon, Amare Bayeh Desta, Etsub Brhanesilassie, Adugna Dufera, and Saira Nawaz

**Response to reviewers: Manuscript Number: PGPH-D-25-00504**

| **Editor Comments** | | |
| --- | --- | --- |
| Q# | Question/comment | Response from authors |
| 1 | Our current Financial Disclosure states, “This study was funded by the United States Agency for International Development (USAID). The funding supported the design, implementation, and analysis of the study. However, the content of this manuscript is solely the responsibility of the authors and does not necessarily reflect the views of USAID or the United States Government.”. However, your funding information on the submission form indicates that you received funding from “President's Malaria Initiative”. Please indicate by return email the full and correct funding information for your study and confirm the order in which funding contributions should appear. Please be sure to indicate whether the funders played any role in the study design, data collection and analysis, decision to publish, or preparation of the manuscript. | Thank you for bringing this to our attention. We confirm that the study was entirely funded by USAID. We apologize for the confusion created. |
| 2 | In the online submission form, you indicated that the data supporting the findings of this study are available upon request from the corresponding author.    All PLOS journals now require all data underlying the findings described in their manuscript to be freely available to other researchers, either  1. In a public repository,  2. Within the manuscript itself, or  3. Uploaded as supplementary information.  This policy applies to all data except where public deposition would breach compliance with the protocol approved by your research ethics board. If your data cannot be made publicly available for ethical or legal reasons (e.g., public availability would compromise patient privacy), please explain your reasons by return email and your exemption request will be escalated to the editor for approval. Your exemption request will be handled independently and will not hold up the peer review process, but will need to be resolved should your manuscript be accepted for publication. One of the Editorial team will then be in touch if there are any issues. | Thank you for bringing this to our attention. For this submission, the data supporting the findings of the study are submitted as an additional supplementary file. |
| 3 | Please provide separate figure files in .tif or .eps format. | Thank you. Figures are submitted separately in .tif format. |
| **Reviewer 1** | | |
| 1 | Abstract: Please make the abstract structured with clear titles for each of the sections: | Thank you for the comment. Our abstract currently addresses the background, objective, methods, key results, and conclusions. However, we did not put subtitles as the journal requirements do not necessitate them. |
| 2 | Abstract: From what I understood in the methods, you did not conduct any surveys but rather interviews with key informants. | Thank you for bringing this to our attention. The confusion arose as the survey was not clearly outlined in the methods section in the previous submission. We extensively revised the current version and clearly mentioned the survey. The data sources are explained in the methods section in the current version as:  Line # 97 to 103: “*Data came from three concurrent components: a quantitative survey, key-informant interviews, and administrative data. The survey quantified the prevalence and perceived importance of implementation factors (contributors and barriers), while the key informant interviews added rich narratives on challenges, lessons learned and successes. Administrative data offered objective indicators and trends that anchored the other findings, such as changes in the availability of laboratories, critical care facilities, and vaccines*.” |
| 3 | Introduction: Could you please refer to the definition of resilience in this introduction. The gist of this manuscript is learning to transform or building back better. This is well explained in this paper: Blanchet K, Nam SL, Ramalingam B, Pozo-Martin F. Governance and Capacity to Manage Resilience of Health Systems: Towards a New Conceptual Framework. Int J Health Policy Manag. 2017 Aug 1;6(8):431-435. doi: 10.15171/ijhpm.2017.36. PMID: 28812842; PMCID: PMC5553211. | We included this definition in the discussion section to explain and discuss how the efforts during the COVID-19 pandemic can be integrated into the broader health system and help improve health system resilience. Here is the excerpt from the discussion:  *Line #583 to 589: “To harness COVID-19 response lessons and strengthen health systems, Ethiopia needs an integrated, context-informed health system framework that reduces siloed approaches, as the WHO building blocks may not fully address health security needs. As Blanchet et al. noted, a health system's resilience is its "capacity to absorb, adapt and transform when exposed to a shock such as a pandemic, natural disaster or armed conflict and still retain the same control over its structure and functions"[38]. Integrating lessons from the COVID-19 response into an adapted framework would enable Ethiopia's health system to fulfill these characteristics and better mitigate future emergencies.”* |
| 4 | Methods: Could you please state when the data were collected. This is important for us to know how sustained the lessons were from the interventions to respond to COVID-19. | Thank you for the comment. We have stated the data collection period in the current version.  Line # 145 to 146: “*Data collection was carried out between August and December 2023.”* |
| 5 | Methods: Line 127 I think you meant study population and not study universe. | Thank you for your question. This section is extensively revised to ensure that the sampling is clear to readers and can be replicated. Here are excerpts in the current version that explain what the target population and study universe mean (Line #105 to 110):  “*We adapted the sampling strategy proposed by Peter et al.[12] to identify populations of actors involved in complex and multi-institutional initiatives. This approach consisted of three key steps: identifying the target population or population universe, defining the source population, and drawing samples from the source population. In this context, the target population was defined as all individuals meeting the inclusion criteria, while the source population referred to the practically accessible segment of this target population who could be feasibly reached for data collection*.” |
| 6 | Methods: Could you please state that the selection of participants was purposive because you describe the rationale (purpose) of selecting the individuals who participated in the study. | Thank you for the comment. The selection of participants actually followed both probability (for quantitative survey) and purposive sampling (key informant interviews). We revised the sampling section to make it clear. The sampling technique is explicitly outlined as follows (line # 117 to 125):  “*Sampling was stratified by both the type of activity conducted and the geographic location to ensure representation across all pillars of the COVID-19 response and the major contextual settings of the country: urban, agrarian, and pastoralist areas. The country was first stratified into these three contexts, from which five regions were randomly selected. Within each selected region, institutions leading the COVID-19 response were identified. From these institutions, all individuals involved in implementing the response who met the inclusion criteria were listed. Individuals were then randomly selected from these lists for a quantitative survey. In cases where only one eligible individual was available, that individual was included (Figure 1). A subset of these quantitative survey participants was subsequently purposively selected for key informant interviews.”* |
| 7 | Methods: Line 134 – What is EPHI? Please write in full at first mention. | Thank you for the question. In the current version, EPHI is fully written out at its first appearance (Line # 194). |
| 8 | Methods: I like the fact that the key informants included covered the broad strategic operations of an emergency response. | Thank you |
| 9 | Methods: Please describe what a woreda is so that an international reader can relate. | Thank you for observing this. We indicated in brackets that woreda means district. |
| 10 | Methods: What informed the design of the guides for the key informant interview guides? Any literature? Framework? Or theory? | Thank you for asking this. All questionnaires are informed by the Consolidated Framework for Implementation Research (CFIR) and the WHO COVID-19 response pillars. This is described in the current submission of the manuscript (Line # 136 to 140):  “*We developed and tested three data collection tools: a structured questionnaire for the quantitative survey, a separate structured questionnaire for individuals working in health facilities, and a semi-structured interview guide for key informant interviews. All tools were developed informed by the Consolidated Framework for Implementation Research (CFIR) domains and designed to cover all WHO pillars of the COVID-19 response.”* |
| 11 | Methods: The use of deductive and inductive approaches to analysis is a good approach to arrive at depth and breadth beyond prescribed frameworks and/ or theories. | Thank you |
| 12 | Methods: The authors do not describe the quantitative data and how they were analyzed. Where were they obtained from? Which indicators were analyzed? | Thank you. We revised and addressed the concerns in the current submission. Here are the sections that describe quantitative data and how they were analyzed, which indicators used, and where they were obtained:  Line # 97 to 103: “*Data came from three concurrent components: a quantitative survey, key-informant interviews, and administrative data. The survey quantified the prevalence and perceived importance of implementation factors (contributors and barriers), while the key informant interviews added rich narratives on challenges, lessons learned and successes. Administrative data offered objective indicators and trends that anchored the other findings, such as changes in the availability of laboratories, critical care facilities, and vaccines*.”  Line # 149 to 154: “*The quantitative survey data underwent descriptive analysis, identifying barriers and facilitators to the implementation of COVID-19 response interventions. This analysis also assessed the level of adoption of guidelines and practices at the health facility level. Guided by CFIR domains and constructs, the analysis examined determinants across the health system, political, economic, social, and technological environments, incorporating both outer and inner CFIR settings. Additionally, trends in key service indicators were computed from the administrative data*.” |
| 13 | Results: I think the results describe many of the response strategies. I am curious as to how the system got better as a result of these response interventions. Could you provide some results that reflect “the system becoming stronger” to say provide vaccination services or maternal and child health services or any other services as a result of interventions during the response. The reason I make this comment is to show how indeed the system learned from the response. | Thank you for raising this important point. We presented the results without adding our own interpretations and without reaching that conclusion. However, we tried to show how the system got better as a result of the response interventions in the discussion section. Here are a few excerpts in the discussion and conclusion section highlighting this:  Line # 560 to 572: “*One key takeaway is the training provided to health workers across various aspects of the response, primarily through an online platform. This platform has established a legacy, serving as a resource for continuous professional development in Ethiopia's health system. Furthermore, the over 3,000 RRTs deployed during the pandemic gained extensive experience in managing various outbreak response activities. If properly documented, these RRTs could be rapidly mobilized in the event of future emergencies, representing a significant asset for the development of the health workforce.*  *The other positive legacy of the COVID-19 response in Ethiopia includes significant developments in laboratory testing, critical care, and digitalization. The expansion of molecular laboratories and the establishment of viral genome sequencing capabilities enhance the country's ability to detect and monitor diseases. In addition, the health system secured critical medical devices and deployed ultracold chain facilities nationwide. Combined with experiences in digitalization, these developments, if integrated into routine healthcare practices, could transform Ethiopia’s health system and ensure resilience.”*  Line # 607 to 612: “*The Ethiopian health system gained valuable experiences and tools during the COVID-19 response, particularly in health workforce development, critical care, digital data solutions, cold chain systems, point of entry, laboratory testing, and community engagement. If these capacities are integrated and institutionalized into the broader health system—particularly within primary health care—and sustained, they have the potential to significantly strengthen Ethiopia's health system, enhance its resilience in daily operations, and provide a robust foundation for preventing and mitigating future risks.”* |
| 14 | Results: I am curious as to the implementation of non-pharmaceutical interventions – movement restrictions, closure of entertainment and religious venues? Were these implemented? It would be good to highlight these as they affected service delivery and use. | Thank you for asking this important question. Yes, we investigated this. However, the result is not part of this manuscript as we are working on another manuscript focusing on health system disruption during the pandemic. Our focus in this manuscript is on the legacy of the pandemic for health system development and health security. |
| 15 | Discussion: Could you discuss how the quantitative results informed the qualitative results? | Thank you for asking this important question. The objectives of conducting a mixed methods in this study were to complement each other. Hence, we believe that the quantitative data complement the qualitative findings as outlined throughout the results sections. As indicated in the methods section, the quantitative survey was done to measure the magnitude of contributors and barriers while the qualitative was to add rich narratives on challenges, lessons and successes.  Line #99 to 103: “*The survey quantified the prevalence and perceived importance of implementation factors (contributors and barriers), while the key informant interviews added rich narratives on challenges, lessons learned and successes. Administrative data offered objective indicators and trends that anchored the other findings, such as changes in the availability of laboratories, critical care facilities, and vaccines.”* |
| 16 | Discussion: Could you also discuss whether/ if there were deliberate strategies to document lessons and learnings? This is important because it is these lessons that strengthen the health system to deliver other services. In addition, if there are deliberate efforts to document lessons, they are more likely to become part of routine service delivery as opposed to adhoc unplanned lessons. | Thank you for the comment. We described this in the current submission in the results section as:  Line # 455 to 457: *“…a national effort documented best practices for sustained use beyond the outbreak. However, these best practices were not done at lower levels.”* |
| 17 | I do not think recall bias as it is applied to epidemiology applies to this study. Recall bias is not forgetting. | Thank you for your valuable comments. We rephrased this and replaced it with a non-technical term.  Line # 662 to 663: *“…the retrospective nature of the information sought may cause inaccurate recall, which could affect the study’s findings.”* |
| 18 | Conclusion: Please do link the conclusions to the results | Thank you. We revised this section to ensure that the conclusion reflects the key findings. Here is an excerpt from the current submission:  Line # 607 to 616: *“The Ethiopian health system gained valuable experiences and tools during the COVID-19 response, particularly in health workforce development, critical care, digital data solutions, cold chain systems, point of entry, laboratory testing, and community engagement. If these capacities are integrated and institutionalized into the broader health system—particularly within primary health care—and sustained, they have the potential to significantly strengthen the health system, enhance its resilience in daily operations, and provide a robust foundation for preventing and mitigating future risks. However, we observed a decline in some practices, particularly in surveillance, laboratory testing, and case management after the initial urgency waned. Our findings thus suggest that while COVID-19 investments expanded life-saving interventions, robust monitoring and follow-up mechanisms are essential to fully integrate and institutionalize many COVID-19 response platforms for future emergencies.”* |
| 19 | References: Please check the references and ensure they align with journal formatting guidance. Especially the abbreviation of Journal names in the reference. | Thank you for your valuable suggestions. We checked each reference and ensured that the citation is in line with the requirements. |
| **Reviewer 2** | | |
| 1 | General comment: The method section was not properly written and the scientific content for a method section was inadequate and not detailed. | Thank you for your comment. We extensively revised the methods section. We hope that the methods in the current submission address journal requirements and are presented in a way that can be replicated by other investigators. |
| 2 | Abstract: Rationale for the study was not stated | Thank you for the comment. In the current submission, we explicitly stated the rationale of the study.  Len # 93 to 95: “*Through this analysis, we aim to contribute to the global evidence base on how health systems can operate and adapt during large-scale public health crises, and leverage these insights to strengthen health systems and health security.”*  Also in the abstract*: “This paper examines Ethiopia's multi-tiered health system response, exploring the strategies, enabling conditions, barriers, and lessons learned.”* |
| 3 | Abstract: What is concurrent nested mixed methods design? There is no such design in Epidemiology. Authors should state the proper study design used | Thank you for the comment. We rephrased the paragraph and addressed the comment as:  Line # 97 to 99: *“We conducted a mixed-methods, cross-sectional study across all tiers of Ethiopia’s health system. Data came from three concurrent components: a quantitative survey, key-informant interviews, and administrative data.”*  Abstract*: “We conducted a mixed-methods, cross-sectional study across all tiers of Ethiopia's health system from August to December 2023.”*  Hope this addresses your concern. |
| 4 | Abstract: Process of analysis should be included in the abstract | Thank you for the comment. Yes, it is included in the current submission as:  “*Quantitative data used descriptive statistics; qualitative data underwent framework analysis, followed by synthesis and triangulation.”* |
| 5 | Introduction: Rationale for the study was not stated | Thank you for the comment. In the current submission, we explicitly stated the rationale of the study.  Len # 93 to 95: “*Through this analysis, we aim to contribute to the global evidence base on how health systems can operate and adapt during large-scale public health crises, and leverage these insights to strengthen health systems and health security.”* |
| 6 | Methods: What is concurrent nested mixed methods design? There is no such design in Epidemiology. Authors should state the proper study design used. Mixed method approach is a data collection method combining both qualitative and quantitative methods of data collection. | Thank you for the comment. We revised the methods and rephrased the design as:  Line # 97 to 99: *“We conducted a mixed-methods, cross-sectional study across all tiers of Ethiopia’s health system. Data came from three concurrent components: a quantitative survey, key-informant interviews, and administrative data.”* |
| 7 | Methods: Move lines 123b – 125 | Thank you for the suggestion. We extensively revised and reorganized the section. |
| 8 | Methods: Study sample lines 126 – 156: this section is confusing and wrongly put together.  i) Authors should state the sampling technique used.  ii) Then re-organize the sampling procedure according to the sampling technique used.  iii) Is this sample for quantitative or qualitative data collection? | Thank you for your valuable suggestions. The methods section is extensively revised in this submission. The sampling techniques and sample size is extensively outlined both for quantitative surveys and key informant interviews. |
| 9 | Methods: Which quantitative data did author collect? Among which group of people? Was it secondary data analysis of records etc? These should be well stated in the methods section. | Thank you for asking this question. In the current submission, the type of data collected, the data source, whether it is secondary or primary, is explicitly outlined. Here are excerpts:  Line # 117 to 125: *Sampling was stratified by both the type of activity conducted and the geographic location to ensure representation across all pillars of the COVID-19 response and the major contextual settings of the country: urban, agrarian, and pastoralist areas. The country was first stratified into these three contexts, from which five regions were randomly selected. Within each selected region, institutions leading the COVID-19 response were identified. From these institutions, all individuals involved in implementing the response who met the inclusion criteria were listed. Individuals* *were then randomly selected from these lists for a quantitative survey. In cases where only one eligible individual was available, that individual was included. A subset of these quantitative survey participants was then purposively selected for key informant interviews.*  Line # 108 to 112: *“…the target population was defined as all individuals meeting the inclusion criteria, while the source population referred to the practically accessible segment of this target population who could be feasibly reached for data collection. Hence, the target population in Ethiopia comprised individuals who had been directly involved in the implementation of COVID-19-related activities for a continuous period of 12 months or more between January 2020 and April 2023.”* |
| 10 | Methods: Authors should state source of questionnaire used for data collection. | Thank you for the comment. In the current submission, we stated and described how the questionnaire used for data collection was developed as:  Line # 136 to 140: “We developed and tested three data collection tools: a structured questionnaire for the quantitative survey, a separate structured questionnaire for individuals working in health facilities, and a semi-structured interview guide for key informant interviews. All tools were developed informed by the Consolidated Framework for Implementation Research (CFIR) domains and designed to cover all WHO pillars of the COVID-19 response.” |
| 11 | How were the data generated? | Thank you for the comment. Primary data were collected using face-to-face interviews, except in a few instances where remote video calls were used. Secondary data were generated from the DHIS2 database of the Ministry of Health, which was set up for data collection, analysis, and storage during the pandemic. Excerpts from the current submission:  Line #v 141 to 145: “*Most interviews were conducted face-to-face; however, a few were conducted virtually via video calls. Virtual interviews were arranged for key individuals who played major roles in the response, were included in the sample, but were unavailable for face-to-face interviews during the data collection period. To ensure data accuracy, all key informant interviews were audio-recorded and transcribed verbatim.”* |
| 12 | What statistical analysis was done on the quantitative data? How were the charts/figure drawn? Trend analysis? | Thank you for asking. The quantitative data was analyzed using descriptive statistics. Trends of key services were also analyzed from administrative data. Here are excerpts from the current submission:  Line # 149 to 154: “*The quantitative survey data underwent descriptive analysis, identifying barriers and facilitators to the implementation of COVID-19 response interventions. This analysis also assessed the level of adoption of guidelines and practices at the health facility level. Guided by CFIR domains and constructs, the analysis examined determinants across the health system, political, economic, social, and technological environments, incorporating both outer and inner CFIR settings. Additionally, trends in key service indicators were computed from the administrative data*.” |
| 13 | Authors should state the Key informant interview guide used for this study | Thank you for asking this. Both quantitative data survey questions and the key informant interview guide are described in this submission. Excerpts:  Line # 138 to 141: “*All tools were developed informed by the Consolidated Framework for Implementation Research (CFIR) domains and designed to cover all WHO pillars of the COVID-19 response. Following tool development, enumerators were recruited and received five days of training, which included pilot testing of the tools outside the study areas.”* |
| 14 | How was the KII guide developed? | The KII guide is developed informed by the Consolidated Framework for Implementation Research (CFIR) domains and designed to cover all WHO pillars of the COVID-19 response. |
| 15 | How many KII was conducted? | Thank you. The KIIs were done mostly face-to-face. In a few instances, the interviews were conducted remotely using video calls. The following is from the current submission. All KIIs were audio recorded.  Line # 141 to 145: “*Most interviews were conducted face-to-face; however, a few were conducted virtually via video calls. Virtual interviews were arranged for key individuals who played major roles in the response, were included in the sample, but were unavailable for face-to-face interviews during the data collection period. To ensure data accuracy, all key informant interviews were audio-recorded and transcribed verbatim.”* |
| 16 | What was the process of the interview? In terms of how many KII were conducted, how long did each interview take? How was the interview recorded? | Thank you. We conducted 80 key informant interviews, and each interview on average took one hour. All interviews were audio-recorded with permission from respondents. Excerpts from the current submission:  Line # 128 to 131: “*Our quantitative survey employed a complex sampling design, which included adjustments for the design effect and potential non-response, to estimate the minimum sample size. This estimation resulted in the inclusion of 952 eligible individuals in the study. Of these, 80 participants were also selected for key informant interviews, with their number determined by data saturation.”* |
